# Association between dietary total antioxidant capacity and semen quality among men attending an infertility clinic: a cross-sectional study

**DOI:** 10.1093/hropen/hoad041

**Published:** 2023-10-31

**Authors:** Dong-Hui Huang, Yi-Xiao Zhang, Xiao-Bin Wang, Ming-Hui Sun, Ren-Hao Guo, Xu Leng, Qiang Du, Hong-Yu Chen, Yu-Xin Nan, Qi-Jun Wu, Bo-Chen Pan, Yu-Hong Zhao

**Affiliations:** Department of Clinical Epidemiology, Shengjing Hospital of China Medical University, Shenyang, China; Liaoning Key Laboratory of Precision Medical Research on Major Chronic Disease, Liaoning, China; Department of Urology, Shengjing Hospital of China Medical University, Shenyang, China; Center for Reproductive Medicine, Shengjing Hospital of China Medical University, Shenyang, China; Department of Clinical Epidemiology, Shengjing Hospital of China Medical University, Shenyang, China; Liaoning Key Laboratory of Precision Medical Research on Major Chronic Disease, Liaoning, China; Center for Reproductive Medicine, Shengjing Hospital of China Medical University, Shenyang, China; Center for Reproductive Medicine, Shengjing Hospital of China Medical University, Shenyang, China; Center for Reproductive Medicine, Shengjing Hospital of China Medical University, Shenyang, China; Department of Clinical Epidemiology, Shengjing Hospital of China Medical University, Shenyang, China; Liaoning Key Laboratory of Precision Medical Research on Major Chronic Disease, Liaoning, China; Department of Clinical Epidemiology, Shengjing Hospital of China Medical University, Shenyang, China; Liaoning Key Laboratory of Precision Medical Research on Major Chronic Disease, Liaoning, China; Department of Clinical Epidemiology, Shengjing Hospital of China Medical University, Shenyang, China; Liaoning Key Laboratory of Precision Medical Research on Major Chronic Disease, Liaoning, China; Department of Obstetrics and Gynecology, Shengjing Hospital of China Medical University, Shenyang, China; NHC Key Laboratory of Advanced Reproductive Medicine and Fertility (China Medical University), National Health Commission, Shenyang, China; Center for Reproductive Medicine, Shengjing Hospital of China Medical University, Shenyang, China; Department of Obstetrics and Gynecology, Shengjing Hospital of China Medical University, Shenyang, China; Department of Clinical Epidemiology, Shengjing Hospital of China Medical University, Shenyang, China; Liaoning Key Laboratory of Precision Medical Research on Major Chronic Disease, Liaoning, China

**Keywords:** sperm, semen quality, dietary total antioxidant capacity, non-enzymatic antioxidant capacity, observational study, clinical study

## Abstract

**STUDY QUESTION:**

Is dietary non-enzymatic antioxidant capacity related to semen quality?

**SUMMARY ANSWER:**

The only statistically significant association of semen quality parameters with dietary total antioxidant capacity (DTAC) detected was an inverse association between DTAC and ejaculate volume.

**WHAT IS KNOWN ALREADY:**

Growing interest exists regarding the role of diet in influencing semen quality. While DTAC is linked to favorable health outcomes, its association with semen quality, especially among men attending infertility clinics, remains understudied.

**STUDY DESIGN, SIZE, DURATION:**

This cross-sectional study was carried out between June and December of 2020. In total, 1715 participants were included in the final analysis.

**PARTICIPANTS/MATERIALS, SETTING, METHODS:**

Men who attended an infertility clinic in China were enrolled. Experienced clinical technicians performed the semen analysis. The DTAC indices included the ferric-reducing ability of plasma, oxygen radical absorbance capacity, total reactive antioxidant potential, and Trolox equivalent antioxidant capacity. The quantile regression model was used for multivariate analysis.

**MAIN RESULTS AND THE ROLE OF CHANCE:**

After adjustment for a variety of confounding variables, a significant inverse association was identified between DTAC and ejaculate volume (β_continuous FRAP_ = −0.015, 95% CI = −0.023, −0.006, β_T3 vs T1_ = −0.193, 95% CI = −0.379, −0.006, *P*_trend_ = 0.007; β_continuous TRAP_ = −0.019, 95% CI = −0.041, 0.002, β_T3 vs T1_ = −0.291, 95% CI = −0.469, −0.112, *P*_trend_ = 0.002). The majority of DTAC indices have no statistically significant association with semen quality parameters.

**LIMITATIONS, REASONS FOR CAUTION:**

We cannot infer causality because of the nature of the cross-sectional study design. The robustness of the conclusion may be compromised by the exactness of non-enzymatic antioxidant capacity estimation.

**WIDER IMPLICATIONS OF THE FINDINGS:**

Our findings demonstrated no association between DTAC indices and semen quality parameters among men attending an infertility clinic, except for ejaculate volume. Even though our findings are mostly non-significant, they contribute novel knowledge to the field of study while also laying the groundwork for future well-designed studies.

**STUDY FUNDING/COMPETING INTEREST(S):**

This work was supported by the JieBangGuaShuai Project of Liaoning Province [grant number 2021JH1/10400050], the Clinical Research Cultivation Project of Shengjing Hospital [grant number M1590], and the Outstanding Scientific Fund of Shengjing Hospital [grant number M1150]. The sponsors had no role in study design, or in the collection, analysis, and interpretation of data, or in the writing of the report, or in the decision to submit the article for publication. There are no conflicts of interest to declare.

**TRIAL REGISTRATION NUMBER:**

N/A.

WHAT DOES THIS MEAN FOR PATIENTS? Male infertility is a global public health issue, and one of its main causes is poor semen quality. The dispute over why semen quality is diminishing has raged for decades, with geographical differences, lifestyle, and environmental factors all being considered. Oxidative stress (which causes tissue damage) is one of the chief mechanisms underlying male infertility and is defined as a disturbance in the balance between production of potentially damaging reactive oxygen species (called free radicals) and antioxidant defenses, i.e. the body’s mechanisms of reducing this stress. Antioxidants are compounds found in foods, particularly certain fruits/vegetables, that scavenge and neutralize the damaging free radicals. A low intake of antioxidant-rich foods, may have a negative impact on semen quality, contributing to reduced male fertility. Because oxidative stress is increasingly recognized to cause sperm damage, there is growing public interest in a possible role of antioxidant-rich diets or antioxidant supplements in improving semen quality and, hence, fertility. The term ‘dietary total antioxidant capacity’ refers to the antioxidant properties derived from the entire diet. We carried out a study of 1715 Chinese men attending an infertility clinic to investigate if there was a link between dietary total antioxidant capacity and semen quality. Our results showed a link between dietary total antioxidant capacity and ejaculate volume but no other features of sperm were linked (e.g. number of sperm, their movement, or structure). However, it is difficult to determine if ejaculate volume alone affects semen quality, and therefore, overall, our study suggests that dietary total antioxidant capacity is not associated with semen quality. Furthermore, because we collected dietary and semen data almost simultaneously, we cannot show a ‘cause and effect’. More well-designed studies into the link between dietary total antioxidant capacity and semen quality are required.

## Introduction

Infertility has been a persistent public health concern in recent decades, with the disease burden projected to have grown in 195 countries and territories between 1990 and 2017 (Sun *et al.*, 2019). According to a recent large-scale demographic analysis based on 204 countries and territories, the global total fertility rate has significantly declined from 4.97 in 1950 to 2.31 in 2019 (GBD 2019 Demographics Collaborators, 2020). Notably, global data suggest that male factors are estimated to account for 20–70% of infertility cases, with an estimated 30 million infertile males worldwide (Agarwal *et al.*, 2015). Furthermore, a review of spatiotemporal trends suggested a deterioration of human semen quality in some specific areas (Auger *et al.*, 2022). Debate regarding the decline in semen quality and the reasons for it has persisted for decades, with geographical differences, lifestyle, and environmental factors all being considered (Virtanen *et al.*, 2017). In males, maintaining a diet rich in antioxidant vitamins, such as vegetables and fruits, has been linked to improved semen quality (Salas-Huetos *et al.*, 2017). A systematic review and meta-analysis of randomized clinical trials also found that some dietary antioxidant supplements could have a beneficial effect on sperm quality parameters (Salas-Huetos *et al.*, 2018).

Assessing the dietary total antioxidant capacity (DTAC) is a comprehensive method for determining the collective antioxidant impact of a diet (Serafini and Del Rio, 2004). Since its introduction, DTAC has been steadily employed in epidemiological studies (Nascimento-Souza *et al.*, 2018). High DTAC has recently been linked to reducing the risk of a variety of health outcomes, including cognitive impairment (Sheng *et al.*, 2022a), cardiovascular disease (Zujko *et al.*, 2022), cancer (Parohan *et al.*, 2019), and all-cause, cardiovascular, or respiratory disease mortality (Sheng *et al.*, 2022b). A review indicated that lifestyle-related oxidative processes might be the primary cause of cellular and tissue damage, which has contributed to the development of non-communicable diseases (Seyedsadjadi and Grant, 2020). However, to our best knowledge, no research has been conducted on the association between DTAC and semen quality, despite the fact that both diet and oxidative stress have been identified as important factors influencing semen quality (Evans *et al.*, 2021; Ferramosca and Zara, 2022).

However, previous studies have mostly focused on the association between antioxidant-rich dietary patterns, dietary supplements, or antioxidant nutrients and semen quality (Salas-Huetos *et al.*, 2017, 2018; Arab *et al.*, 2018; De Cosmi *et al.*, 2021), rather than DTAC that measures quality of the entire diet (Salari-Moghaddam *et al.*, 2022). Meanwhile, the applicability of the aforementioned evidence may vary across different countries owing to local variations in dietary habits. Hence, the current study aimed to evaluate the association between DTAC and semen quality in Chinese men.

## Materials and methods

### Participants

The research methodology has been previously reported (Liu *et al.*, 2021, 2022). Briefly, this cross-sectional study was carried out between June and December 2020. Men who attended the infertility clinic at Shengjing Hospital of China Medical University were enrolled. Male participants were included based on the following criteria: provided informed consent to participate in the study; being 18 years of age or older; and being a first-time visitor who had not undergone any medical treatment, including medication, surgery, or combination therapy, for more than 3 months. Men who: refused to participate in the study; did not fully complete the questionnaire; or had previously been diagnosed with infertility at another hospital were excluded.

**Figure 1.**
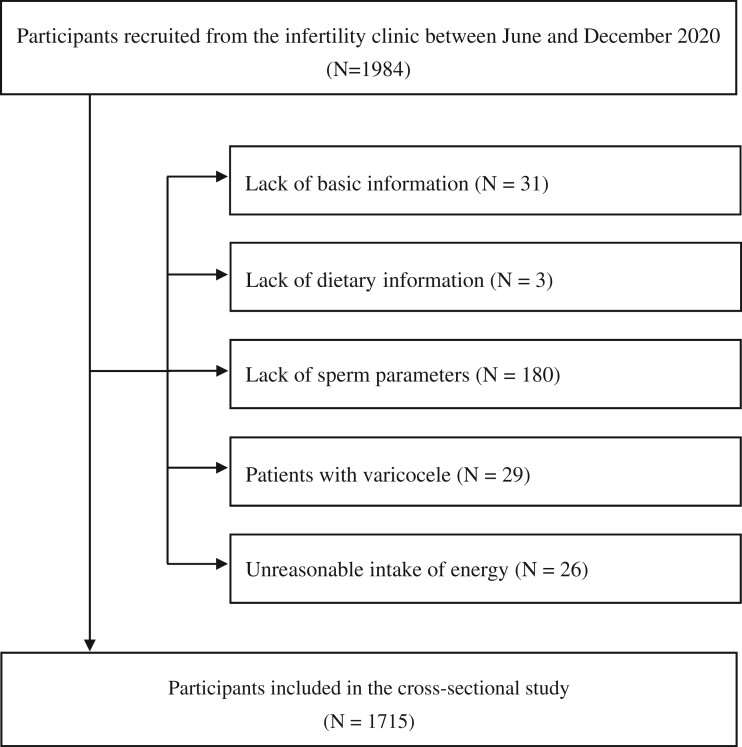
Flow diagram for selecting participants in a study of dietary total antioxidant capacity and semen quality among Chinese men attending an infertility clinic.

As shown in Fig. 1, a total of 1984 male participants were recruited. After excluding participants with missing basic information (n = 31), missing dietary information (n = 3), missing semen parameters (n = 180), a history of varicocele (n = 29), and unexplained total energy intake (<800 or >6000 kcal/day) (n = 26) (Yuan *et al.*, 2021), the final analysis included 1715 participants. PASS (Power Analysis and Sample Size) software, version 11.0 (NCSS, LLC. Kaysville, UT, USA) determined that this number matched the required sample size of 1645 men.

### Ethical approval

All participants signed informed consent forms at the time of study enrollment. This study received approval by the ethics committee of Shengjing Hospital of China Medical University (2017PS190K).

### Data collection

Data were collected using relevant questionnaires, including a demographic and lifestyle questionnaire, a physical activity questionnaire, and a food frequency questionnaire (FFQ). We collected information such as region (Liaoning/other provinces), age (years), annual family income (RMB; thousand yuan), education level (junior secondary or below, senior high school/technical secondary school, and university/junior college or above), occupational status (employed/unemployed), alcohol intake status (yes/no), smoking status (yes/no), and use of nutritional supplements (yes/no) by using a demographic and lifestyle questionnaire. To determine physical activity levels, we employed the identical questionnaire as used in the China Kadoorie Biobank study (Du *et al.*, 2013). The FFQ with 110 food items was used to assess dietary intake (Cui *et al.*, 2023a). The auditor quickly checked for omissions and errors after participants completed the on-site self-administered questionnaire. Every participant underwent a physical examination and semen analysis. A trained assistant measured the participant’s height and weight during the physical examination.

### Semen analysis

Semen analyses were all performed by experienced clinical technicians. Prior to semen collection, the males were instructed to abstain for 3–7 days. Semen samples were collected by individuals in a designated private room adjacent to the laboratory, through masturbation into a sterile plastic container, abstaining from condom use. A WLJY9000 computer-assisted sperm analyzer (Beijing Weili New Century Science & Tech. Dev. Co. Ltd, Beijing, China) was used to evaluate the collected semen samples. The key detection metrics were semen volume, total sperm count, sperm concentration, progressive motility, total sperm motility, and sperm morphology. Total motility was defined as the sum of progressive and non-progressive motility. A semen smear was made and stained using the Papanicolaou method, and the sperm morphology was assessed by examining the semen smear under an optical microscope. The World Health Organization laboratory manual for the examination and processing of human semen (5th Edition, 2010) was employed to determine the quality of the semen (WHO, 2010). External quality control was implemented throughout the study (Li *et al.*, 2022; Zhao *et al.*, 2023).

### Dietary intake assessment

A previously validated FFQ tailored exclusively for the population of Northeast China was employed to acquire dietary information (Cui *et al.*, 2023a). Participants were asked to report on their average frequency of consumption of each FFQ food item in the previous year. The portion size (g/time) derived from the weighed diet records was used to translate these frequencies to grams of food consumed (g) (Cui *et al.*, 2023a). The daily nutrient intake was then calculated by multiplying the mass of each food (g/day) by the nutrient content (per 100 g of food) in the Chinese Food Composition Tables (Yang *et al.*, 2018b).

### Dietary total antioxidant capacity

The ferric-reducing ability of plasma (FRAP) (Carlsen *et al.*, 2010), the oxygen radical absorbance capacity (U.S. Department of Agriculture, Agricultural Research Service, 2010), the total radical-trapping antioxidant parameter (TRAP) (Pellegrini *et al.*, 2003, 2006), and the Trolox equivalent antioxidant capacity (TEAC) (Pellegrini *et al.*, 2003, 2006) were used for determining the DTAC value. Each FFQ 110 food item was cross-referenced with its corresponding DTAC database. If an FFQ item did not correspond to one in the database, a proxy estimate based on the average of similar items was employed. Each participant’s DTAC was computed by multiplying the daily foods consumed by the relevant DTAC value per food portion and summing these values. The DTAC estimates did not account for nutritional supplements, including vitamin and mineral supplements, cod liver oil, or DHA supplements, as well as ginseng supplement.

### Other variables

Physical activity was quantified by multiplying each type of activity by the corresponding metabolic equivalent of task (MET) intensity values (Ainsworth *et al.*, 2011). The BMI was computed by dividing weight in kilograms by height in meters squared (kg/m^2^).

The eating habits section of the FFQ was utilized to gather information on recent dietary changes and cooking methods. Participants were asked if they had made any recent dietary changes, with four options available: ‘None’, ‘From 3 years ago’, ‘From 1 to 2 years ago’, and ‘From this year’. These responses were translated to ‘yes’ or ‘no’ during the analysis to indicate the dietary change. Participants were asked how frequently they eat meat, vegetables, and seafood that had been steamed, stewed, broiled, deep-fried, stir-fried, or eaten raw. These frequencies were then converted to weekly times, and the values for each cooking type were tallied.

### Statistical analysis

Demographic data were categorized into DTAC tertiles. If the continuous variables had a normal distribution, the means with SDs were reported and compared using one-way ANOVA. If the continuous variables were not normally distributed, they were expressed as medians with interquartile ranges (IQRs) and compared using the Kruskal–Wallis test. The Chi-square test was used to compare categorical variables presented as frequencies (%). The residual method was applied to adjust for energy in the DTAC indices and other dietary factors. The DTAC indices were analyzed as either continuous variables or tertiles. The linear trend across increasing tertiles was analyzed using the median value of each tertile as a continuous variable. The quantile (median) regression model was used for the multivariable analysis (Nassan *et al.*, 2020). Furthermore, we used restricted cubic spline (RCS) with three knots positioned at the 5th, 50th, and 95th percentiles of the distribution to investigate potential nonlinear associations between the DTAC indices and semen quality parameters.

Potential confounding variables in the multivariable model were energy intake (continuous, kcal/day), region (Liaoning/other provinces), age (continuous, years), BMI (continuous, kg/m^2^), fiber intake (continuous, g/day), annual family income (continuous, thousand yuan), physical activity (continuous, MET/h/week), abstinence time (continuous, days), smoking status (yes/no), alcohol drinking status (yes/no), education level (junior secondary or below, senior high school/technical secondary school, and university/junior college or above), occupational status (employed/unemployed), nutritional supplements use (yes/no), recent dietary changes (yes/no), and frequencies of cooking methods (continuous, times/week).

Subgroup analyses were performed depending on age (<32 and ≥32 years), smoking status (yes/no), nutritional supplements use (yes/no), and recent dietary changes (yes/no). The multiplicative and additive interactions of DTAC indices with the previously mentioned factors were examined. In addition, we conducted several sensitivity analyses. First, an analysis was carried out to rule out the possibility of normozoospermic men skewing the current data. Second, as is common in semen quality studies, a multivariate linear regression model with a log-transformed dependent variable was used. Third, we performed a sensitivity analysis based on the tertiles of semen quality parameters to help interpret the results. Finally, to evaluate the impact of missing values on the robustness of the present findings, we employed multiple imputation to address missing values (Austin *et al.*, 2021). SAS software, version 9.4 (SAS Institute, Cary, NC, USA), was utilized for all analyses. In all statistical tests, a *P*-value of <0.05 was considered statistically significant.

## Results

### Participant characteristics and dietary intake

**Figure 2.**
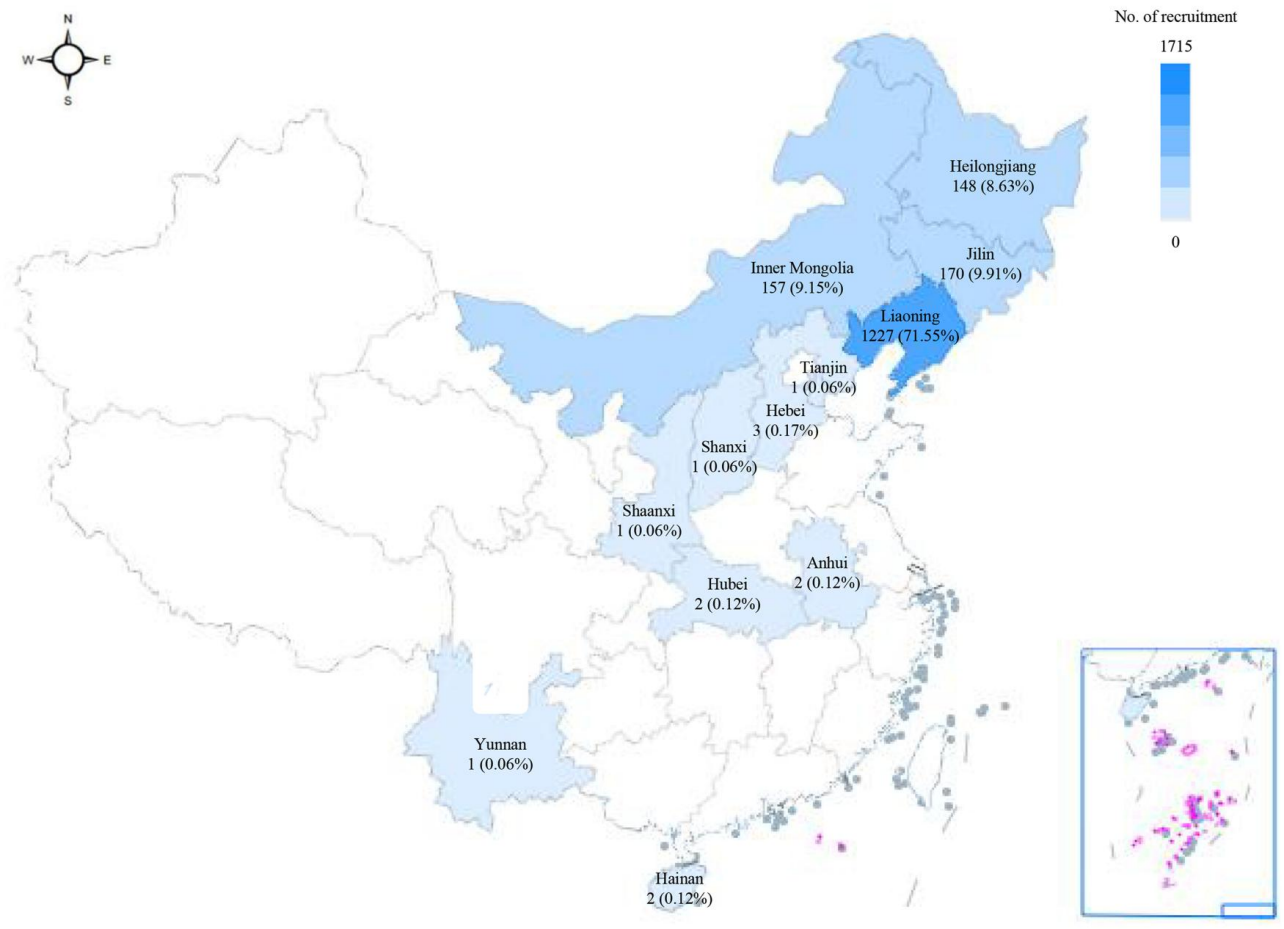
Regional distribution within China of participants in the study.

The characteristics of all participants are shown in Table 1. The median age of the participants was 32.0 years, and 63.9% had a junior college/university degree or above. An estimated 71.6% of the participants originated from Liaoning Province (Fig. 2). Supplementary Table S1 shows the distribution of DTAC and semen quality parameters. The median FRAP (mmol/day) with IQR was 6.7 (5.1, 10.2). The median total sperm count (×10^6^/ml) was 161.0 (86.9, 261.8). The median percentage of normal sperm morphology was 4.0 (3.0, 7.0). In Supplementary Table S2, participant characteristics are shown by DTAC tertiles. Except for region, abstinence time, sperm concentration, and normal sperm morphology, all variables were significantly associated with at least one DTAC index (*P *<* *0.05).

**Table 1.** Baseline demographic, reproductive, and nutritional characteristics of the infertile participants.

Variables	All participants
**No. of participants**	1715
**Region**	
Liaoning	1227 (71.6)
Other provinces	488 (28.4)
**Age (**years**)**	32.0 (30.0, 35.0)
**BMI (**kg/m^2^**)**	26.0 (23.4, 28.7)
**Occupation (**n, %**)**	
Employed	1248 (72.8)
Unemployed	467 (27.2)
**Education (**n, %**)**	
Junior secondary or below	375 (21.9)
Senior high school/technical secondary school	244 (14.2)
Junior college/university or above	1096 (63.9)
**Annual family income (**RMB, 10,000 yuan**)**	8.0 (5.0, 10.0)
**Drinking (**n, %**)**	682 (39.8)
**Smoking (**n, %**)**	856 (49.9)
**Nutritional supplements use (**n, %**)**	252 (14.7)
**No dietary change (**n, %**)**	1330 (77.6)
**Physical activity (**MET/h/week**)**	133.6 (99.3, 223.5)
**Abstinence time (**days**)**	4.0 (3.0, 5.0)
**Deviations from reference semen values**	
Ejaculate volume <1.5 ml	1637 (95.5)
Total sperm count <39 × 10^6^/ml	1556 (90.7)
Sperm concentration <15 × 10^6^/ml	1538 (89.7)
Progressive motility <32%	787 (45.9)
Total motility <40%	776 (45.3)
Normal sperm morphology <4%	1171 (68.3)
**Abnormal semen parameters**	
Asthenozoospermia	551 (32.1)
Teratozoospermia	148 (8.6)
Oligozoospermia	12 (0.7)
Asthenoteratozoospermia	245 (14.3)
Oligoasthenozoospermia	59 (3.4)
Oligospermia	10 (0.6)
Oligoasthenoteratozoospermia	107 (6.2)
Normozoospermic men (%)	583 (34.0)
**Food groups**	
Staple food (g/day)	653.4 (538.7, 743.6)
Dairy (g/day)	89.4 (43.5, 168.4)
Meat (g/day)	110.1 (80.2, 137.2)
Egg (g/day)	31.1 (18.6, 48.7)
Fish (g/day)	21.7 (12.6, 30.6)
Vegetable (g/day)	234.9 (178.5, 313.1)
Bean (g/day)	83.1 (55.5, 124.0)
Pickled food (g/day)	16.6 (9.7, 24.0)
Fruit (g/day)	138.3 (87.4, 209.3)
Snacks (g/day)	48.1 (31.4, 67.9)
Tea/coffee (g/day)	59.5 (18.9, 194.2)
Alcoholic beverages (g/day)	59.5 (16.1, 222.2)
Nonalcoholic beverage (g/day)	48.7 (21.8, 114.4)
**Nutrients**	
Energy (kcal/day)	1703.9 (1398.2, 2100.3)
Carbohydrate (%E)	57.2 (52.3, 61.6)
Fat (%E)	25.0 (22.0, 28.0)
Protein (%E)	16.5 (15.2, 17.8)
Fiber (g/day)	17.1 (14.5, 20.2)
Vitamin A (µgRE)	550.4 (394.4, 778.6)
Vitamin C (mg/day)	104.1 (79.2, 136.4)
Vitamin E (mg/day)	15.4 (12.4, 19.4)
Carotenoid (µg/day)	2194.7 (1465.1, 3039.1)
Flavonoids (mg/day)	81.8 (60.6, 109.0)
Magnesium (mg/day)	307.6 (281.4, 336.0)
Zinc (mg/day)	11.6 (10.7, 12.6)
Selenium (µg/day)	41.5 (36.2, 47.8)
**Cooking methods**	
Deep-frying (times/week)	1.2 (0.6, 1.8)
Stewing (times/week)	5.6 (3.5, 8.1)
Broiling (times/week)	1.2 (0.6, 1.8)
Stir-frying (times/week)	9.5 (7.5, 12.0)
Steaming (times/week)	0.6 (0.0, 1.2)
Raw (times/week)	1.0 (0.6, 2.5)

MET, metabolic equivalent task; No, number.

Data are presented as a median (*P*_25_, *P*_75_) or as a count (%).

The residual method was used to adjust the intake variables for energy intake.

### The association between DTAC indices and semen quality parameters

Table 2 presents the energy-adjusted quantile regression coefficients with 95% CIs for semen parameters concerning the DTAC indices. The DTAC indices demonstrated an association with ejaculate volume (β_continuous FRAP_ = −0.013, 95% CI = −0.023, −0.002, β_T3 vs T1_ = −0.323, 95% CI = −0.545, −0.101, *P*_trend_ = 0.006; β_continuous TRAP_ = −0.023, 95% CI = −0.046, 0.000, β_T3 vs T1_ = −0.500, 95% CI = −0.716, −0.284, *P*_trend_ < 0.001), and progressive motility (β_continuous TRAP_ = −0.269, 95% CI = −0.636, 0.099, β_T3 vs T1_ = −3.274, 95% CI = −5.966, −0.581, *P*_trend_ = 0.021).

**Table 2.** Energy-adjusted quantile regression coefficients with 95% CI for semen parameters in relation to the DTAC indices.

Variables	**Ejaculate volume (**ml**)**	**Total sperm count (**10^6^/ml**)**	**Sperm concentration (**10^6^/ml**)**	**Progressive motility (**%**)**	**Total motility (**%**)**	**Normal sperm morphology (**%**)**
**FRAP (**mmol/day**)**						
T1	0 (Ref)	0 (Ref)	0 (Ref)	0 (Ref)	0 (Ref)	0 (Ref)
T2	−0.027 (−0.259, 0.241)	−0.825 (−21.709, 20.059)	2.512 (−3.034, 8.058)	−0.960 (−3.652, 1.731)	0.986 (−2.785, 4.757)	0.000 (−0.000, 0.000)
T3	−0.323 (−0.545, −0.101)	−12.226 (−30.480 6.028)	2.390 (−2.865, 7.645)	−0.868 (−3.334, 1.598)	−0.070 (−2.721, 2.581)	0.000 (−0.000, 0.000)
*P*-trend	0.006	0.191	0.520	0.687	0.921	1.000
Continuous	−0.013 (−0.023, −0.002)	−0.158 (−1.088, 0.772)	0.061 (−0.248, 0.370)	−0.051 (−0.209, 0.108)	−0.038 (−0.190, 0.115)	0.000 (−0.000, 0.000)
**T-ORAC (**μmol TE/day**)**						
T1	0 (Ref)	0 (Ref)	0 (Ref)	0 (Ref)	0 (Ref)	0 (Ref)
T2	−0.200 (−0.439, 0.039)	3.852 (−20.777, 28.482)	−1.192 (−6.944, 4.561)	−0.069 (−2.780, 2.643)	0.550 (−2.200, 3.300)	0.000 (−0.000, 0.000)
T3	−0.200 (−0.412, 0.012)	5.047 (−12.775, 22.869)	0.807 (−4.015, 5.628)	−0.370 (−2.682, 1.942)	−0.265 (−3.599, 3.068)	0.000 (−0.000, 0.000)
*P*-trend	0.033	0.575	0.862	0.890	0.805	–
Continuous	0.000 (−0.000, 0.000)	0.000 (−0.000, 0.000)	−0.000 (−0.000, 0.000)	−0.000 (−0.000, 0.000)	0.000 (−0.000, 0.000)	0.000 (−0.000, 0.000)
**TRAP (**mmol TE/day**)**						
T1	0 (Ref)	0 (Ref)	0 (Ref)	0 (Ref)	0 (Ref)	0 (Ref)
T2	−0.300 (−0.514, −0.086)	3.947 (−12.725, 20.619)	2.415 (−2.516, 7.345)	−0.771 (−3.394, 1.851)	−0.607 (−3.887, 2.673)	0.000 (−0.000, 0.000)
T3	−0.500 (−0.716, −0.284)	−16.025 (−34.677, 2.627)	−1.856 (−7.090, 3.378)	−3.274 (−5.966, −0.581)	−2.747 (−5.909, 0.415)	0.000 (−0.000, 0.000)
*P*-trend	< 0.001	0.047	0.340	0.021	0.100	1.000
Continuous	−0.023 (−0.046, 0.000)	−0.036 (−3.062, 2.991)	0.245 (−0.506, 0.997)	−0.269 (−0.636, 0.099)	−0.197 (−0.629, 0.235)	0.000 (−0.000, 0.000)
**TEAC (**mmol TE/day**)**						
T1	0 (Ref)	0 (Ref)	0 (Ref)	0 (Ref)	0 (Ref)	0 (Ref)
T2	0.000 (−0.285, 0.285)	10.278 (−7.775, 28.331)	1.879 (−3.827, 7.585)	−1.722 (−4.581, 1.137)	−2.315 (−5.970, 1.340)	0.000 (−0.000, 0.000)
T3	−0.200 (−0.419, 0.019)	−10.494 (−28.293, 7.304)	−1.073 (−6.412, 4.266)	−2.860 (−5.601, −0.120)	−2.300 (−5.377, 0.777)	0.000 (−0.000, 0.000)
*P*-trend	0.075	0.129	0.676	0.056	0.075	1.000
Continuous	−0.035 (−0.073, 0.002)	−1.006 (−4.841, 2.830)	0.383 (−0.716, 1.481)	−0.410 (−0.978, 0.158)	−0.332 (−0.910, 0.246)	0.000 (−0.000, 0.000)
**H-ORAC (**μmol TE/day**)**						
T1	0 (Ref)	0 (Ref)	0 (Ref)	0 (Ref)	0 (Ref)	0 (Ref)
T2	0.100 (−0.149, 0.349)	13.380 (−7.086, 33.846)	−2.064 (−7.182, 3.054)	2.027 (−0.683, 4.736)	1.839 (−1.137, 4.815)	0.000 (−0.000, 0.000)
T3	0.000 (−0.177, 0.177)	6.033 (−11.081, 23.148)	0.204 (−4.968, 5.377)	−0.274 (−2.458, 1.909)	0.059 (−2.821, 2.939)	0.000 (−0.000, 0.000)
*P*-trend	1.000	0.487	0.762	0.885	0.504	–
Continuous	0.000 (−0.000, 0.000)	0.000 (−0.001, 0.001)	−0.000 (−0.000, 0.000)	−0.000 (−0.000, 0.000)	0.000 (−0.000, 0.000)	0.000 (−0.000, 0.000)
**L-ORAC (**μmol TE/day**)**						
T1	0 (Ref)	0 (Ref)	0 (Ref)	0 (Ref)	0 (Ref)	0 (Ref)
T2	−0.200 (−0.409, 0.009)	18.967 (0.699, 37.234)	2.760 (−3.161, 8.681)	3.005 (0.845, 5.165)	3.586 (0.504, 6.668)	0.000 (−0.000, 0.000)
T3	−0.200 (−0.406, 0.006)	8.127 (−9.250, 25.504)	1.302 (−3.480, 6.083)	−0.591 (−2.894, 1.712)	−1.194 (−4.058, 1.671)	0.000 (−0.000, 0.000)
*P*-trend	0.049	0.108	0.425	0.438	0.507	–
Continuous	−0.000 (−0.000, 0.000)	0.000 (−0.000, 0.001)	0.000 (−0.000, 0.000)	0.000 (−0.000, 0.000)	0.000 (−0.000, 0.000)	0.000 (−0.000, 0.000)
**TP (**mg GAE/day**)**						
T1	0 (Ref)	0 (Ref)	0 (Ref)	0 (Ref)	0 (Ref)	0 (Ref)
T2	−0.200 (−0.424, 0.024)	0.060 (−22.805, 22.925)	−1.332 (−6.775, 4.110)	0.745 (−1.927, 3.417)	1.429 (−1.552, 4.411)	0.000 (−0.000, 0.000)
T3	−0.200 (−0.402, 0.002)	5.936 (−12.301, 24.172)	−1.999 (−7.695, 3.697)	−0.029 (−2.571, 2.513)	0.463 (−2.644, 3.570)	0.000 (−0.000, 0.000)
*P*-trend	1.000	0.567	0.432	0.945	0.655	–
Continuous	0.000 (−0.000, 0.000)	0.002 (−0.007, 0.010)	−0.002 (−0.004, 0.001)	−0.000 (−0.001, 0.001)	0.000 (−0.001, 0.002)	0.000 (−0.000, 0.000)

DTAC, dietary total antioxidant capacity; FRAP, ferric-reducing ability of plasma; GAE, gallic acid equivalents; H-ORAC, hydrophilic oxygen radical absorbance capacity; L-ORAC, lipophilic oxygen radical absorbance capacity; Ref, reference; T, tertile; T-ORAC, total oxygen radical absorbance capacity; TE, Trolox equivalents; TEAC, Trolox equivalent antioxidant capacity; TP, total phenolics; TRAP, total radical-trapping antioxidant parameter.

As shown in Table 3, multivariable-adjusted quantile regression revealed that DTAC was significantly associated with ejaculate volume (β_continuous FRAP_ = −0.015, 95% CI = −0.023, −0.006, β_T3 vs T1_ = −0.193, 95% CI = −0.379, −0.006, *P*_trend_ = 0.007; β_continuous TRAP_ = −0.019, 95% CI = −0.041, 0.002, β_T3 vs T1_ = −0.291, 95% CI = −0.469, −0.112, *P*_trend_ = 0.002). The association between TRAP and total motility was marginally significant (β_continuous TRAP_ = −0.137, 95% CI = −0.578, 0.303, β_T3 vs T1_ = −3.277, 95% CI = −6.287, −0.266, *P*_trend_ = 0.055). The statistically significant quantile regression results are presented in Supplementary Fig. S1.

**Table 3.** Multivariate-adjusted quantile regression coefficients with 95% CI for semen parameters in relation to the DTAC indices.

Variables	**Ejaculate volume (**ml**)**	**Total sperm count (**10^6^/ml**)**	**Sperm concentration (**10^6^/ml**)**	**Progressive motility (**%**)**	**Total motility (**%**)**	**Normal sperm morphology (**%**)**
**FRAP (**mmol/day**)**						
T1	0 (Ref)	0 (Ref)	0 (Ref)	0 (Ref)	0 (Ref)	0 (Ref)
T2	0.007 (−0.208, 0.222)	0.691 (−18.955, 20.337)	−0.084 (−5.877, 5.708)	0.183 (−2.725, 3.092)	0.247 (−3.584, 4.077)	0.072 (−0.257, 0.400)
T3	−0.193 (−0.379, −0.006)	−2.180 (−20.149, 15.789)	0.683 (−4.714, 6.081)	0.127 (−2.462, 2.717)	0.113 (−2.959, 3.186)	0.035 (−0.258, 0.328)
*P*-trend	0.007	0.796	0.776	0.945	0.998	0.874
Continuous	−0.015 (−0.023, −0.006)	−0.024 (−0.742, 0.695)	0.079 (−0.212, 0.369)	−0.031 (−0.149, 0.086)	−0.038 (−0.201, 0.125)	−0.002 (−0.016, 0.012)
**T-ORAC (**μmol TE/day**)**						
T1	0 (Ref)	0 (Ref)	0 (Ref)	0 (Ref)	0 (Ref)	0 (Ref)
T2	0.085 (−0.107, 0.276)	−3.571 (−21.725, 14.582)	−2.170 (−7.393, 3.052)	0.529 (−1.948, 3.005)	1.170 (−1.870, 4.210)	−0.056 (−0.323, 0.212)
T3	−0.110 (−0.285, 0.066)	−0.707 (−18.130, 16.716)	−0.556 (−5.768, 4.657)	0.104 (−2.351, 2.558)	0.279 (−2.851, 3.409)	−0.072 (−0.335, 0.191)
*P*-trend	0.172	0.862	0.698	0.941	0.683	0.572
Continuous	−0.000 (−0.000, 0.000)	−0.000 (−0.000, 0.000)	−0.000 (−0.000, 0.000)	0.000 (−0.000, 0.000)	0.000 (−0.000, 0.000)	−0.000 (−0.000, 0.000)
**TRAP (**mmol TE/day**)**						
T1	0 (Ref)	0 (Ref)	0 (Ref)	0 (Ref)	0 (Ref)	0 (Ref)
T2	−0.108 (−0.317, 0.100)	3.842 (−12.654, 20.337)	1.994 (−2.973, 6.961)	0.112 (−2.243, 2.467)	−0.222 (−3.257, 2.813)	−0.044 (−0.336, 0.248)
T3	−0.291 (−0.469, −0.112)	−6.616 (−24.385, 11.153)	−0.333 (−5.530, 4.865)	−2.185 (−4.761, 0.390)	−3.277 (−6.287, −0.266)	0.029 (−0.241, 0.300)
*P*-trend	0.002	0.234	0.697	0.100	0.055	0.832
Continuous	−0.019 (−0.041, 0.002)	0.588 (−1.273, 2.450)	0.271 (−0.512, 1.055)	−0.181 (−0.506, 0.145)	−0.137 (−0.578, 0.303)	0.011 (−0.032, 0.053)
**TEAC (**mmol TE/day**)**						
T1	0 (Ref)	0 (Ref)	0 (Ref)	0 (Ref)	0 (Ref)	0 (Ref)
T2	0.009 (−0.183, 0.202)	10.200 (−6.486, 26.886)	2.007 (−3.095, 7.109)	−1.866 (−4.293, 0.560)	−1.961 (−4.842, 0.921)	−0.091 (−0.369, 0.188)
T3	−0.149 (−0.331, 0.032)	−4.994 (−22.919, 12.931)	−0.026 (−5.380, 5.327)	−2.328 (−4.888, 0.232)	−2.496 (−5.389, 0.397)	0.001 (−0.290, 0.291)
*P*-trend	0.094	0.391	0.912	0.101	0.077	0.816
Continuous	−0.034 (−0.068, −0.000)	1.047 (−1.714, 3.807)	0.514 (−0.625, 1.652)	−0.306 (−0.787, 0.174)	−0.243 (−0.905, 0.419)	0.015 (−0.047, 0.077)
**H-ORAC (**μmol TE/day**)**						
T1	0 (Ref)	0 (Ref)	0 (Ref)	0 (Ref)	0 (Ref)	0 (Ref)
T2	0.067 (−0.106, 0.241)	4.108 (−12.750, 20.966)	−1.279 (−5.854, 3.297)	2.033 (−0.114, 4.179)	3.276 (0.627, 5.924)	−0.213 (−0.456, 0.030)
T3	−0.061 (−0.241, 0.119)	−0.640 (−19.264, 17.983)	−0.711 (−5.987, 4.565)	0.840 (−1.734, 3.414)	1.491 (−1.539, 4.522)	−0.090 (−0.392, 0.212)
*P*-trend	0.526	0.669	0.735	0.411	0.317	0.570
Continuous	−0.000 (−0.000, 0.000)	−0.000 (−0.000, 0.000)	−0.000 (−0.000, 0.000)	0.000 (−0.000, 0.000)	0.000 (−0.000, 0.000)	−0.000 (−0.000, 0.000)
**L-ORAC (**μmol TE/day**)**						
T1	0 (Ref)	0 (Ref)	0 (Ref)	0 (Ref)	0 (Ref)	0 (Ref)
T2	−0.022 (−0.194, 0.151)	13.091 (−6.240, 32.422)	1.952 (−3.308, 7.213)	2.712 (0.497, 4.926)	3.979 (1.084, 6.873)	0.123 (−0.198, 0.445)
T3	−0.108 (−0.310, 0.093)	0.624 (−16.594, 17.842)	0.515 (−4.985, 6.014)	−0.606 (−3.253, 2.042)	−1.498 (−4.928, 1.932)	−0.135 (−0.417, 0.147)
*P*-trend	0.207	0.657	0.612	0.320	0.420	0.738
Continuous	−0.000 (−0.000, 0.000)	0.000 (−0.000, 0.000)	−0.000 (−0.000, 0.000)	0.000 (−0.000, 0.000)	0.000 (−0.000, 0.000)	−0.000 (−0.000, 0.000)
**TP (**mg GAE/day**)**						
T1	0 (Ref)	0 (Ref)	0 (Ref)	0 (Ref)	0 (Ref)	0 (Ref)
T2	−0.035 (−0.227, 0.157)	−4.796 (−22.816, 13.225)	−1.415 (−6.657, 3.827)	1.382 (−1.152, 3.916)	1.729 (−1.439, 4.898)	−0.155 (−0.431, 0.121)
T3	−0.143 (−0.321, 0.035)	−0.715 (−18.794, 17.364)	−2.803 (−8.112, 2.506)	0.244 (−2.280, 2.769)	−0.109 (−3.195, 2.977)	−0.097 (−0.372, 0.177)
*P*-trend	0.082	0.983	0.289	0.675	0.668	0.536
Continuous	−0.000 (−0.000, 0.000)	−0.004 (−0.011, 0.004)	−0.002 (−0.004, 0.000)	0.000 (−0.001, 0.001)	−0.000 (−0.001, 0.001)	−0.000 (−0.000, 0.000)

DTAC, dietary total antioxidant capacity; FRAP, ferric-reducing ability of plasma; GAE, gallic acid equivalents; H-ORAC, hydrophilic oxygen radical absorbance capacity; L-ORAC, lipophilic oxygen radical absorbance capacity; Ref, reference; T, tertile; T-ORAC, total oxygen radical absorbance capacity; TE, Trolox equivalents; TEAC, Trolox equivalent antioxidant capacity; TP, total phenolics; TRAP, total radical-trapping antioxidant parameter.

The model was adjusted for total energy intake (kcal/day), region (Liaoning/other provinces), age (years), BMI (kg/m^2^), fiber intake (g/day), annual family income (RMB; thousand yuan), physical activity (MET/h/week), abstinence time (days), smoking (yes/no), drinking (yes/no), education (junior secondary or below, senior high school/technical secondary school, and junior college/university or above), occupation (employed/unemployed), nutritional supplements use (yes/no), dietary change (yes/no), and cooking methods (times/week).

**Figure 3.**
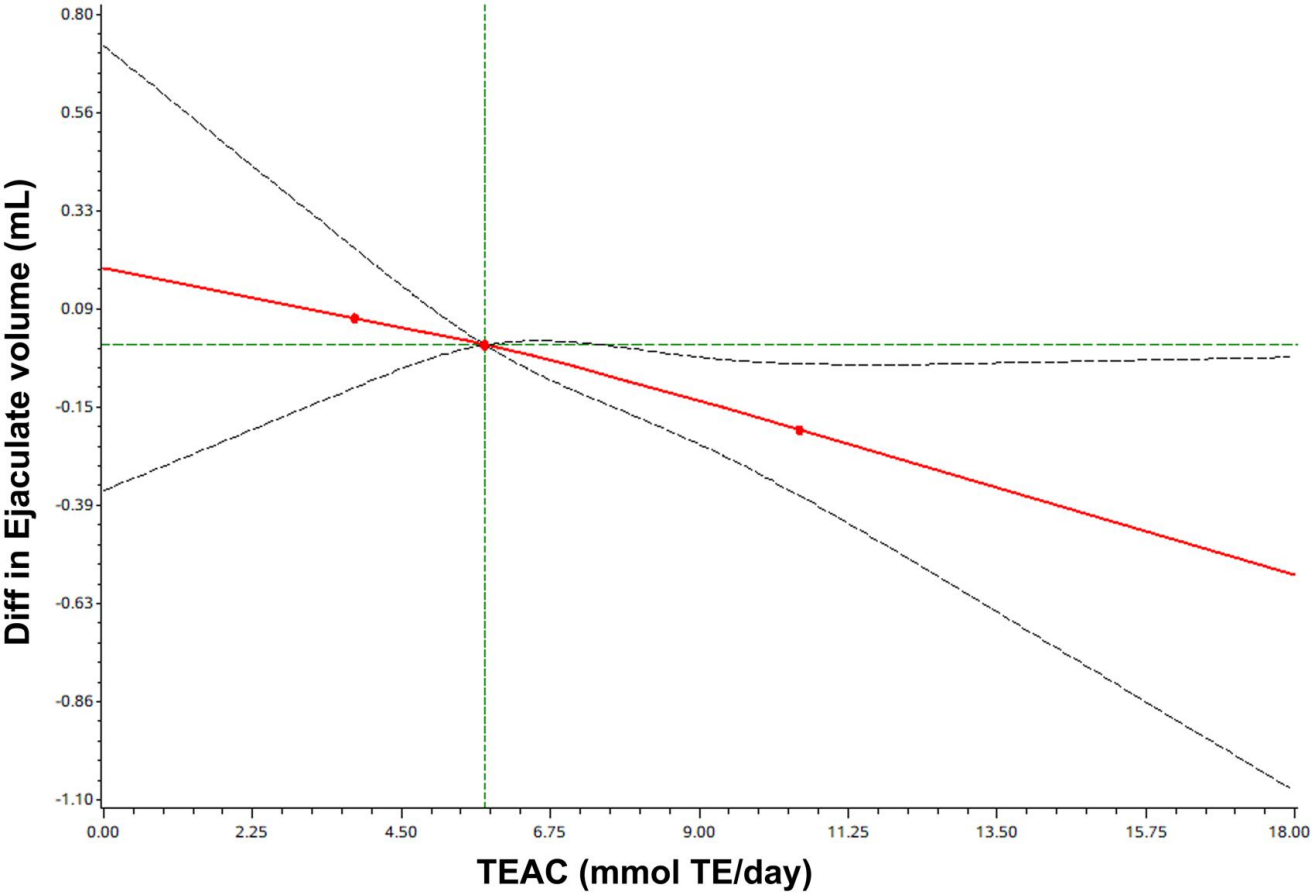
**The dose–response association between Trolox equivalent antioxidant capacity and ejaculate volume in infertile Chinese men.** Diff, difference; TE, Trolox equivalents; TEAC, Trolox equivalent antioxidant capacity. Difference in ejaculate volume where the reference value for TEAC is median.

With the exception of increased TEAC demonstrating a linear link with lower ejaculate volume (*P*_overall_ = 0.038, *P*_nonlinear_ = 0.823) (Fig. 3), all other RCS results were deemed non-significant after multivariable adjustments (*P*_overall_ > 0.05). The RCS curves for FRAP and semen quality parameters are depicted in Supplementary Fig. S2.

### Subgroup, interaction, and sensitivity analyses

**Figure 4.**
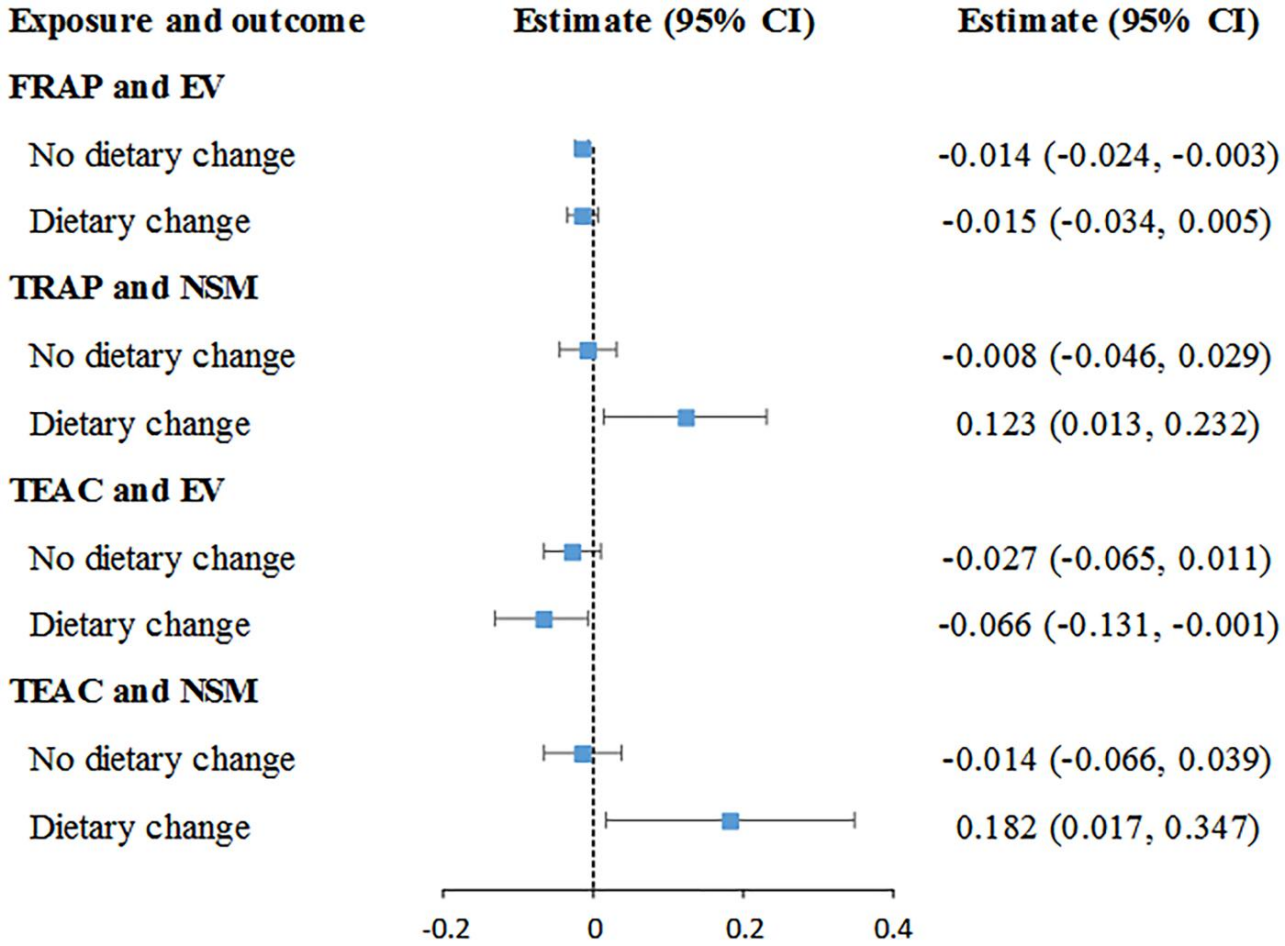
**The statistically significant results of the dietary change subgroup analysis.** EV, ejaculate volume; FRAP, ferric-reducing ability of plasma; NSM, normal sperm morphology; TEAC, Trolox equivalent antioxidant capacity; TRAP, total radical-trapping antioxidant parameter.

Results of the subgroup analyses are shown in Supplementary Table S3. FRAP was significantly linked with ejaculate volume in the ≥32 years old subgroup (β_continuous FRAP_ = −0.014, 95% CI = −0.025, −0.002), smoking subgroup (β_continuous FRAP_ = −0.015, 95% CI = −0.027, −0.003), no nutritional supplement use subgroup (β_continuous FRAP_ = −0.013, 95% CI = −0.023, −0.004), and no dietary change group (β_continuous FRAP_ = −0.014, 95% CI = −0.024, −0.003) (Supplementary Fig. S3). Furthermore, upon dietary change subgroup analyses, a clear association was observed between TRAP and normal sperm morphology (β_continuous TRAP_ = 0.123, 95% CI = 0.013, 0.232); TEAC was associated with ejaculate volume (β_continuous TEAC_ = −0.066, 95% CI = −0.131, −0.001) and normal sperm morphology (β_continuous TEAC_ = 0.182, 95% CI = 0.017, 0.347) (Fig. 4). In addition, TRAP was associated with progressive motility in nonsmokers (β_continuous TRAP_ = −0.402, 95% CI = −0.784, −0.019). Other subgroup analyses yielded no statistically significant results.

Both additive and multiplicative interaction models indicated a modifying effect of dietary change on the relation between DTAC and semen quality parameters (Relative excess risk due to interaction (RERI)_FRAP and Ejaculate volume_ = −1.616, 95% CI = −3.053, −0.179, *P*_multiplicative interaction_ = 0.025; RERI_TRAP and Ejaculate volume_ = −1.320, 95% CI = −2.610, −0.029, *P*_multiplicative interaction_ = 0.049; RERI_FRAP and Normal sperm morphology_ = 0.616, 95% CI = 0.092, 1.140, *P*_multiplicative interaction_ = 0.031) (Supplementary Table S4).

In linear regression models (Table 4), DTAC indices were found to be related to ejaculate volume (β_continuous TRAP_ = −0.006, SE = 0.003, β_T3 vs T1_ = −0.084, SE = 0.026, *P*_trend_ = 0.002; β_continuous TEAC_ = −0.011, SE = 0.005, β_T3 vs T1_ = −0.052, SE = 0.027, *P*_trend_ = 0.037). After sensitivity analysis excluding normozoospermic individuals, DTAC indices were still associated with ejaculate volume (β_continuous FRAP_ = −0.011, 95% CI = −0.022, −0.000, β_T3 vs T1_ = −0.306, 95% CI = −0.546, −0.066, *P*_trend_ = 0.007; β_continuous TRAP_ = −0.033, 95% CI = −0.067, 0.000, β_T3 vs T1_ = −0.256, 95% CI = −0.478, −0.035, *P*_trend_ = 0.002), as shown in Supplementary Table S5. Moreover, sensitivity analysis based on semen parameter tertiles demonstrated a link between TRAP and ejaculative volume (Ejaculative volume_T3 vs T1_: odds ratio (OR)_T3 vs T1_ = 0.637, 95% CI: 0.470, 0.861, *P*_trend_ = 0.003) (Supplementary Table S6). Finally, as shown in Supplementary Table S7, the multiple imputation analysis also found that DTAC was associated with ejaculate volume (β_continuous FRAP_ = −0.009, 95% CI = −0.018, −0.001, β_T3 vs T1_ = −0.193, 95% CI = −0.366, −0.021, *P*_trend_ = 0.049; β_continuous TRAP_ = −0.026, 95% CI = −0.047, −0.006, β_T3 vs T1_ = −0.310, 95% CI = −0.477, −0.143, *P*_trend_< 0.001; β_continuous TEAC_ = −0.042, 95% CI = −0.073, −0.011, β_T3 vs T1_ = −0.225, 95% CI = −0.397, −0.053, *P*_trend_ = 0.008).

**Table 4.** Multivariate-adjusted linear regression coefficients with standard errors for semen parameters in relation to the DTAC indices.

Variables	**Ejaculate volume (**ml**)**	**Total sperm count (**10^6^/ml**)**	**Sperm concentration (**10^6^/ml**)**	**Progressive motility (**%**)**	**Progressive motility (**%**)**	**Normal sperm morphology (**%**)**
**FRAP (**mmol/day**)**						
T1	Ref	Ref	Ref	Ref	Ref	Ref
T2	−0.026 (0.027)	−0.035 (0.067)	−0.009 (0.064)	0.013 (0.045)	−0.008 (0.044)	0.035 (0.039)
T3	−0.039 (0.028)	0.003 (0.067)	0.042 (0.064)	0.076 (0.045)	0.063 (0.044)	0.033 (0.039)
*P*-trend	0.2066	0.7972	0.4174	0.0705	0.0843	0.5344
Continuous	−0.002 (0.001)	−0.002 (0.003)	0.001 (0.003)	0.001 (0.002)	0.000 (0.002)	0.001 (0.002)
**T-ORAC (**μmol TE/day**)**						
T1	Ref	Ref	Ref	Ref	Ref	Ref
T2	−0.010 (0.027)	0.006 (0.066)	0.016 (0.063)	−0.037 (0.045)	−0.021 (0.043)	−0.036 (0.038)
T3	−0.059 (0.026)	−0.020 (0.064)	0.039 (0.061)	−0.037 (0.043)	−0.037 (0.042)	−0.042 (0.037)
*P*-trend	0.0529	0.8201	0.5588	0.3410	0.3873	0.2289
Continuous	−0.000 (0.000)	0.000 (0.000)	0.000 (0.000)	−0.000 (0.000)	−0.000 (0.000)	−0.000 (0.000)
**TRAP (**mmol TE/day**)**						
T1	Ref	Ref	Ref	Ref	Ref	Ref
T2	−0.047 (0.026)	0.047 (0.064)	0.094 (0.061)	0.009 (0.043)	0.013 (0.042)	0.033 (0.037)
T3	−0.084 (0.026)	−0.098 (0.064)	−0.013 (0.062)	−0.038 (0.044)	−0.034 (0.042)	0.024 (0.038)
*P*-trend	0.0017	0.0729	0.5943	0.3188	0.3454	0.5982
Continuous	−0.006 (0.003)	−0.000 (0.008)	0.005 (0.008)	−0.001 (0.005)	0.001 (0.005)	0.005 (0.005)
**TEAC (**mmol TE/day**)**						
T1	Ref	Ref	Ref	Ref	Ref	Ref
T2	0.002 (0.027)	0.018 (0.065)	0.016 (0.062)	−0.042 (0.044)	−0.051 (0.042)	−0.025 (0.038)
T3	−0.052 (0.027)	−0.070 (0.066)	−0.018 (0.064)	−0.039 (0.045)	−0.037 (0.043)	0.003 (0.039)
*P*-trend	0.0368	0.2374	0.7334	0.4353	0.4620	0.8521
Continuous	−0.011 (0.005)	−0.004 (0.012)	0.007 (0.012)	−0.002 (0.008)	0.000 (0.008)	0.008 (0.007)
**H-ORAC (**μmol TE/day**)**						
T1	Ref	Ref	Ref	Ref	Ref	Ref
T2	0.002 (0.026)	0.068 (0.063)	0.067 (0.060)	0.009 (0.042)	0.026 (0.041)	−0.031 (0.036)
T3	−0.037 (0.026)	−0.003 (0.063)	0.034 (0.060)	−0.043 (0.043)	−0.036 (0.041)	−0.024 (0.037)
*P*-trend	0.1784	0.9388	0.5123	0.3512	0.4425	0.4826
Continuous	−0.000 (0.000)	0.000 (0.000)	0.000 (0.000)	−0.000 (0.000)	−0.000 (0.000)	−0.000 (0.000)
**L-ORAC (**μmol TE/day**)**						
T1	Ref	Ref	Ref	Ref	Ref	Ref
T2	−0.014 (0.028)	0.137 (0.067)	0.150 (0.064)	0.091 (0.045)	0.099 (0.044)	0.045 (0.039)
T3	−0.051 (0.028)	−0.010 (0.067)	0.041 (0.064)	−0.052 (0.045)	−0.052 (0.044)	−0.062 (0.039)
*P*-trend	0.1320	0.3756	0.1162	0.8525	0.7702	0.6093
Continuous	−0.000 (0.000)	0.000 (0.000)	0.000 (0.000)	−0.000 (0.000)	−0.000 (0.000)	−0.000 (0.000)
**TP (**mg GAE/day**)**						
T1	Ref	Ref	Ref	Ref	Ref	Ref
T2	−0.021 (0.026)	−0.049 (0.063)	−0.028 (0.060)	−0.013 (0.043)	−0.002 (0.041)	−0.037 (0.037)
T3	−0.035 (0.026)	−0.001 (0.064)	0.035 (0.061)	−0.027 (0.043)	−0.029 (0.042)	−0.041 (0.037)
*P*-trend	0.1726	0.9279	0.6253	0.5313	0.5154	0.2523
Continuous	−0.000 (0.000)	−0.000 (0.000)	0.000 (0.000)	−0.000 (0.000)	−0.000 (0.000)	−0.000 (0.000)

DTAC, dietary total antioxidant capacity; FRAP, ferric-reducing ability of plasma; GAE, gallic acid equivalents; H-ORAC, hydrophilic oxygen radical absorbance capacity; L-ORAC, lipophilic oxygen radical absorbance capacity; Ref, reference; T, tertile; T-ORAC, total oxygen radical absorbance capacity; TE, Trolox equivalents; TEAC, Trolox equivalent antioxidant capacity; TP, total phenolics; TRAP, total radical-trapping antioxidant parameter.

The model was adjusted for total energy intake (kcal/day), region (Liaoning/other provinces), age (years), BMI (kg/m^2^), fiber intake (g/day), annual family income (RMB; thousand yuan), physical activity (MET/h/week), abstinence time (days), smoking (yes/no), drinking (yes/no), education (junior secondary or below, senior high school/technical secondary school, and junior college/university or above), occupation (employed/unemployed), nutritional supplements use (yes/no), dietary change (yes/no), and cooking methods (times/week).

## Discussion

To the best of our knowledge, this is the first study to examine the relationship between DTAC and semen quality. Given the lack of a perfect assay, the current study applied a variety of commonly used DTAC indices rather than a single index, which might compensate for each other’s shortcomings and result in more accurate DTAC estimates. We found that the crude model related DTAC indices to ejaculate volume and progressive motility. An inverse association between DTAC and ejaculate volume was consistently observed after multivariable adjustment. Furthermore, following multiple sensitivity analyses, the relationship between DTAC and ejaculate volume remained robust. However, ejaculate volume alone is insufficient to predict semen quality, and given that the majority of our findings are non-significant, our study is more likely to conclude that the influence of DTAC on semen quality appears to be insignificant.

Biologically, oxidative stress is currently recognized as one of the primary mechanisms underlying male infertility (Cassina *et al.*, 2015; Bisht *et al.*, 2017; Barati *et al.*, 2020). Antioxidants, which serve as ‘scavengers’ of reactive oxygen species (ROS), appear to be promising therapeutic approaches for reversing the negative impact of high ROS levels on semen parameters (Bisht *et al.*, 2017; Barati *et al.*, 2020). In recent years, the role of antioxidant-rich diets or antioxidant supplements in male fertility has gained considerable attention (Salas-Huetos *et al.*, 2017; Nassan *et al.*, 2018; Ferramosca and Zara, 2022; Zafar *et al.*, 2023). For example, according to a review, a low intake of antioxidant-rich foods, such as fruits/vegetables or products with antioxidant potential, may have a negative impact on semen quality, contributing to poorer male fertility (Skoracka *et al.*, 2020). Moreover, antioxidant supplements, such as coenzyme Q10, appear to improve sperm quality by lowering testicular oxidative stress and sperm DNA fragmentation (Lucignani *et al.*, 2022). In contrast, despite a plausible theoretical foundation, some studies have produced statistically non-significant results. For instance, in a recent case–control study, our colleagues found no significant link between DTAC and asthenozoospermia (Huang *et al.*, 2023), and the present cross-sectional study on DTAC and semen quality parameters also yielded statistically non-significant findings. However, it should be noted that the two studies mentioned above, as well as many others on dietary antioxidants, are observational, thus limiting their ability to infer causality. In fact, the use of dietary antioxidants for male fertility has been impeded by an absence of high-quality evidence. For example, a 2018 review found little high-quality evidence to determine whether antioxidant consumption helps or harms infertile couples (Smits *et al.*, 2018). Furthermore, a Cochrane systematic review on antioxidants for male subfertility, updated in 2022, revealed that the pooled results for total sperm motility, progressive sperm motility, and concentration at 3, 6, and 9 months were unreliable because of extremely high heterogeneity in each analysis (de Ligny *et al.*, 2022). Therefore, additional high-quality randomized controlled trials are needed to determine factors such as the type of antioxidant utilized, the dose, the time to be administered, the duration of treatment, and the cost of antioxidants in male infertility (Dias *et al.*, 2020; Agarwal *et al.*, 2021).

There are several potential explanations for our findings. First, the quality of semen may be affected by a variety of factors, including monogenic factors (Houston *et al.*, 2021), ambient air pollution (Xu *et al.*, 2023), cigarette smoking (Sharma *et al.*, 2016), as well as dietary factors (Salas-Huetos *et al.*, 2017). If the oxidative stress only plays a secondary role in the pathogenesis, augmenting antioxidant defense alone may be insufficient for disease treatment or prevention (Forman and Zhang, 2021). Second, dietary antioxidants may be a double-edged sword. On the one hand, there is substantial evidence that antioxidant supplements, fruits, vegetables, and other antioxidant-rich products can help improve semen quality (Nassan *et al.*, 2018; Skoracka *et al.*, 2020; Zafar *et al.*, 2023). On the other hand, maintaining a balance between ROS levels and antioxidant defense appears to be more critical (Chianese and Pierantoni, 2021) because a certain level of ROS is necessary for sperm function, and excessive antioxidants may inhibit the normal signaling pathways activated by ROS molecules, causing harm to the male reproductive system (Dias *et al.*, 2020).

Another consideration is that the antioxidant defense-inducing compounds may not achieve effective concentrations *in vivo* (Forman and Zhang, 2021). Antioxidants may exhibit dose-dependent effects. One review showed that many antioxidants could act as pro-oxidants at high doses, increasing oxidative stress and causing toxicity (Yang *et al.*, 2018a). Dietary natural polyphenols were shown to have either positive or negative effects on sperm mitochondrial function, depending on the concentration (Ferramosca and Zara, 2022). An investigation of 16 individual compounds also revealed that certain polyphenols, at high concentrations, could act as pro-oxidants or alkylating agents in spermatozoa, resulting in severe negative effects on the biological competence of these cells, with only resveratrol, genistein and 2,2’,4,4’-tetrahydroxydiphenyl maintaining an antioxidant function and having no adverse effects on sperm when the dose was <100 μM (Aitken *et al.*, 2016). Furthermore, in a study of the modulation of plant polyphenols on human sperm mitochondrial respiratory efficiency, researchers found that quercetin, naringin, genistein, luteolin, and resveratrol could significantly improve mitochondrial respiratory efficiency at 0.1 nM but significantly decreased it at 10 nM (Ferramosca *et al.*, 2021). Collectively, it could be said that the effect of natural antioxidants on spermatozoa may be concentration dependent.

On the other hand, variability in semen quality could also be one of the factors influencing our findings. To clarify this issue, we compared the variability of semen parameters in the current study to that of similar Chinese studies (Wang *et al.*, 2015; Yang *et al.*, 2017; Miao *et al.*, 2021), and we found that most semen parameters had roughly identical variability, with even lower variability in our data on normal sperm morphology and slightly higher variability in sperm count. Furthermore, a previous study that involved 10 laboratories assessing intra- and inter-individual variability in human semen quality concluded that external quality control schemes were necessary for maintaining the quality of semen analysis (Auger *et al.*, 2000). In our study, experienced technicians performed external quality control as part of the Chinese Medical Association Society of Reproductive Medicine’s nationwide quality control program on semen analysis (Li *et al.*, 2022; Zhao *et al.*, 2023). Our thorough external quality control ensures the quality of semen analysis and, to some extent, minimizes the variability of semen quality parameter measures.

Finally, dietary constancy may be another essential factor to consider in the analysis of association between DTAC and semen quality because sperm collected during enrollment was produced over a 74-day period, whereas we employed a validated FFQ to follow dietary intake over the past 12 months (Cui *et al.*, 2023a). However, according to a systematic review, FFQs with a 12-month dietary recall interval exhibit superior FFQ reproducibility when compared to <12 months (Cui *et al.*, 2021), indicating that our FFQ approach for assessing dietary intake may be stable. Moreover, to eliminate the influence of dietary changes on the conclusion, we included dietary change during the preceding year as an adjustment variable, as in previous studies (Huang *et al.*, 2023; Zhao *et al.*, 2023). In addition, we conducted a subgroup analysis and an interaction analysis based on the dietary change status. Finally, we found that dietary changes might act as an effect modifier in the association between DTAC and specific semen parameters. Except for the above results, most of the subgroup and interaction analysis results were non-significant, corresponding to the primary findings. Therefore, it is speculated that dietary changes were unlikely to affect the conclusion of this study.

The current study possesses certain strengths. First, as mentioned above, this is the first study to investigate DTAC in relation to semen parameters. Second, quantile regression analyses between seven DTAC indices and six semen parameters, as well as numerous subgroup analyses, interaction analyses, and sensitivity analyses, were performed. These analyses may contribute to a better understanding of the association, especially between certain DTAC indices and specific semen quality parameters, while also boosting the robustness and generalizability of the conclusion.

Nevertheless, some limitations should be noted. First, the cross-sectional nature of our study limits our ability to establish causality. Second, although a previous systematic review on the validity of the FFQ indicated that it was suitable to estimate overall dietary intake in a nutritional epidemiological study (Cui *et al.*, 2023b), the FFQ employed is known to be subject to recall bias. Third, data on a previous coronavirus disease 2019 (COVID-19) diagnosis, a known risk factor for sperm parameters, was not collected in this study (Li *et al.*, 2020). However, according to the official data for 2020 (available at http://www.nhc.gov.cn/jkj/s3578/202103/f1a448b7df7d4760976fea6d55834966.shtml), the incidence of COVID-19 in China was low throughout the study year, with an estimated 6.2 per 100 000 people. Furthermore, while participants came from various provinces, most of them lived in Liaoning Province when they enrolled, and according to government data, Liaoning province was free of COVID-19 from June to December 2020 (available at https://wsjk.ln.gov.cn/wsjk/zfxxgk/fdzdgknr/tfggsj/bdc99f4d-2.shtml). In addition, everyone who visited Shengjing Hospital during the study period had to be screened for COVID-19 and then transferred to a fever clinic if they tested positive. As a result, despite not specifically excluding them, the current study included nearly no patients with COVID-19. Fourth, there are some limitations to the DTAC estimate. First of all, nutritional supplements and oils were not included in the DTAC calculation owing to the limited number of FFQ food items and the difficulties in quantifying them. Moreover, these DTAC indices may not accurately reflect the antioxidant status *in vivo* since assessing the *in vitro* antioxidant capacity of isolated compounds, beverages, or extracts is insufficient for estimating their antioxidant effects *in-vivo*, according to a recent review (Mota *et al.*, 2023). In addition, we had to rely on international databases because we lacked DTAC values for local food items. This measurement bias could not be completely avoided despite the use of several DTAC indices and controlling for diet-related confounding factors to compensate for indices estimation shortcomings. Furthermore, food processing may affect DTAC estimation accuracy since thermal and nonthermal operations alter the phenolic antioxidants in fruits, vegetables, and grains (Nayak *et al.*, 2015). Finally, although the confounding factors of cooking methods were taken into account in the current analysis, there were some unmeasurable food processing procedures, such as blanching and drying, that could influence the DTAC estimates. Fifth, while the variability of semen parameters was low in our study, and external quality control was performed to help ensure this, slight fluctuations may still limit our ability to discover the association between DTAC and semen quality. Variability of semen parameters is a common issue in similar studies that should be noted and addressed in future study.

## Conclusion

Our findings demonstrated no association between DTAC indices and semen quality parameters among men attending an infertility clinic, with the exception of ejaculate volume. Even though our findings do not imply a substantial association, they contribute novel knowledge to the field of study while also laying the groundwork for future well designed studies.

## Supplementary Material

hoad041_Supplementary_Tables

hoad041_Supplementary_Figures

## Data Availability

The data that support the findings of our study are available from the corresponding author upon reasonable request.
