# Supplementary material for: Association between dietary total antioxidant capacity and semen quality among men attending an infertility clinic: a cross-sectional study
Source: Hum Reprod Open. 2023 Oct 31;2023(4):hoad041. doi: 10.1093/hropen/hoad041 (PMC10639034; doi:10.1093/hropen/hoad041)
Supplement: hoad041_Supplementary_Tables [file hoad041_supplementary_tables.docx]

**Supplementary Table S1** Distribution of dietary total antioxidant capacity and semen quality parameters.

| **Variables** | **Median (*P*_25_, *P*_75_)** | **Mean****±SD** | **Range (Min, Max)** |
| --- | --- | --- | --- |
| **Exposure** |  |  |  |
| FRAP (mmol/day) | 6.7 (5.1, 10.2) | 9.3±8.3 | 79.3 (-5.1, 74.2) |
| T-ORAC (μmol TE/day) | 139905.6 (99481.5, 150856.7) | 127471.0±31353.4 | 209036.5 (30720.4, 239756.9) |
| TRAP (mmol TE/day) | 6.0 (5.1, 7.3) | 6.8±3.3 | 36.3 (-2.9, 33.4) |
| TEAC (mmol TE/day) | 5.8 (5.0, 6.7) | 6.2±2.2 | 21.8 (-0.9, 20.9) |
| H-ORAC (μmol TE/day) | 66677.0 (57442.3, 73407.6) | 66055.7±13491.8 | 131768.9 (17549.2, 149318.1) |
| L-ORAC (μmol TE/day) | 72764.9 (39711.4, 76812.1) | 59473.9±21512.0 | 89977.4 (-4193.5, 85783.9) |
| TP (mg GAE/day) | 5212.4 (4527.9, 5728.2) | 5136.8±905.7 | 6533.7 (2204.1, 8737.8) |
| **Outcome** |  |  |  |
| Ejaculate volume (mL) | 3.2 (2.4, 4.2) | 3.5±1.5 | 11.5 (0.5, 12.0) |
| Total sperm count (10^6^ / mL) | 161.0 (86.9, 261.8) | 189.0±141.2 | 1535.9 (0.3, 1536.2) |
| Sperm concentration (10^6^ / mL) | 48.7 (28.7, 75.9) | 56.9±41.2 | 419.0 (0.1, 419.1) |
| Progressive motility (%) | 30.4 (19.1, 41.3) | 30.6±15.3 | 81.7 (0.0, 81.7) |
| Total motility (%) | 37.1 (23.9, 51.4) | 37.9±18.5 | 94.5 (0.0, 94.5) |
| Normal sperm morphology (%) | 4.0 (3.0, 7.0) | 4.9±3.1 | 21.0 (0.0, 21.0) |

FRAP: Ferric reducing antioxidant power; GAE: Gallic acid equivalents; H-ORAC: Hydrophilic oxygen radical absorbance capacity; L-ORAC: Lipophilic oxygen radical absorbance capacity; Max: Maximum value; Min: Minimum value; SD: Standard deviation; T-ORAC: Total oxygen radical absorbance capacity; TE: Trolox equivalents; TEAC: Trolox equivalent antioxidant capacity; TP: Total phenolics; TRAP: Total radical-trapping antioxidant parameter.

**Supplementary Table S2** Participant characteristics based on energy-adjusted dietary total antioxidant capacity.

| **Variables** | **FRAP** (mmol/d) | | **T-ORAC** (μmol TE/d) | | **TRAP** (mmol TE/d) | | **TEAC** (mmol TE/d) | | **H-ORAC** (μmol TE/d) | | **L-ORAC** (μmol TE/d) | | **TP** (mg GAE/d) | |
| --- | --- | --- | --- | --- | --- | --- | --- | --- | --- | --- | --- | --- | --- | --- |
|  | T1 (<5.7) | T3 (≥8.4) | T1 (< 110884.9) | T3 (≥147591.5) | T1 (<5.4) | T3 (≥6.8) | T1 (<5.3) | T3 (≥6.3) | T1 (< 61405.4) | T3 (≥71219.4) | T1 (< 42449.6) | T3 (≥75689.7) | T1 (< 4796.9) | T3 (≥5545.2) |
| **No.** | 573 | 571 | 573 | 571 | 573 | 571 | 573 | 571 | 573 | 571 | 573 | 571 | 573 | 571 |
| **Region** |  |  |  |  |  |  |  |  |  |  |  |  |  |  |
| Liaoning | 411 (71.7) | 400 (70.1) | 416 (72.6) | 391 (68.5) | 415 (72.4) | 394 (69.0) | 411 (71.7) | 400 (70.1) | 406 (70.9) | 406 (71.1) | 413 (72.1) | 392 (68.7) | 412 (71.9) | 392 (68.7) |
| Other provinces | 162 (28.3) | 171 (29.9) | 157 (27.4) | 180 (31.5) | 158 (27.6) | 177 (31.0) | 162 (28.3) | 171 (29.9) | 167 (29.1) | 165 (28.9) | 160 (27.9) | 179 (31.3) | 161 (28.1) | 179 (31.3) |
| **Age (**years**)** | 33.0 (31.0, 37.0) | 31.0 (29.0, 34.0) ^†^ | 32.0 (30.0, 35.0) | 33.0 (30.0, 36.0) | 33.0 (31.0, 37.0) | 32.0 (29.0, 34.0) ^†^ | 33.0 (31.0, 37.0) | 32.0 (29.0, 34.0) ^†^ | 32.0 (30.0, 36.0) | 32.0 (30.0, 35.0) | 32.0 (29.0, 35.0) | 33.0 (30.0, 36.0) ^†^ | 32.0 (29.0, 35.0) | 33.0 (30.0, 36.0) ^†^ |
| **BMI (**kg/m^2^**)** | 26.5 (24.2, 29.4) | 25.7 (23.0, 28.1) ^†^ | 25.6 (23.1, 28.4) | 26.3 (23.8, 29.4) ^†^ | 26.3 (23.7, 29.3) | 25.9 (23.2, 28.4) ^†^ | 26.3 (23.9, 29.2) | 25.9 (23.2, 28.4) ^†^ | 26.0 (23.4, 28.7) | 26.3 (23.8, 29.3) | 25.4 (22.7, 28.1) | 26.3 (24.0, 29.3) ^†^ | 26.0 (23.2, 28.7) | 26.3 (23.8, 29.4) ^†^ |
| **Occupation (***n*, %**)** |  |  |  |  |  |  |  |  |  |  |  |  |  |  |
| Employed | 410 (71.6) | 427 (74.8) | 404 (70.5) | 425 (74.4) | 425 (74.2) | 408 (71.5) | 415 (72.4) | 419 (73.4) | 402 (70.2) | 422 (73.9) | 402 (70.2) | 423 (74.1) | 395 (68.9) | 424 (74.3) ^‡^ |
| Unemployed | 163 (28.4) | 144 (25.2) | 169 (29.5) | 146 (25.6) | 148 (25.8) | 163 (28.5) | 158 (27.6) | 152 (26.6) | 171 (29.8) | 149 (26.1) | 171 (29.8) | 148 (25.9) | 178 (31.1) | 147 (25.7) |
| **Education (***n*, %**)** |  |  |  |  |  |  |  |  |  |  |  |  |  |  |
| Junior secondary or below | 87 (15.2) | 149 (26.1) ^‡^ | 147 (25.7) | 113 (19.8) ^‡^ | 88 (15.4) | 146 (25.6) ^‡^ | 96 (16.8) | 140 (24.5) ^‡^ | 128 (22.3) | 115 (20.1) | 165 (28.8) | 106 (18.5) ^‡^ | 145 (25.3) | 103 (18.0) ^‡^ |
| Senior high school | 75 (13.1) | 79 (13.8) | 101 (17.6) | 63 (11.0) | 69 (12.0) | 92 (16.1) | 73 (12.7) | 93 (16.3) | 93 (16.2) | 65 (11.4) | 101 (17.6) | 65 (11.4) | 108 (18.9) | 64 (11.2) |
| University or above | 411 (71.7) | 343 (60.1) | 325 (56.7) | 395 (69.2) | 416 (72.6) | 333 (58.3) | 404 (70.5) | 338 (59.2) | 352 (61.4) | 391 (68.5) | 307 (53.6) | 400 (70.1) | 320 (55.9) | 404 (70.8) |
| **Income (**Ten thousand yuan**)** | 10.0 (5.0, 12.0) | 8.0 (5.0, 10.0) ^†^ | 8.0 (5.0, 10.0) | 8.0 (5.0, 10.0) ^†^ | 10.0 (5.0, 12.0) | 8.0 (5.0, 10.0) ^†^ | 10.0 (5.0, 12.0) | 8.0 (5.0, 10.0) ^†^ | 8.0 (5.0, 10.0) | 8.0 (5.0, 10.0) | 7.0 (5.0, 10.0) | 10.0 (5.0, 10.0) ^†^ | 8.0 (5.0, 10.0) | 8.0 (5.0, 10.0) |
| **Drinking (***n*, %**)** | 256 (44.7) | 202 (35.4) ^‡^ | 233 (40.7) | 248 (43.4) ^‡^ | 204 (35.6) | 268 (46.9) ^‡^ | 218 (38.1) | 256 (44.8) ^‡^ | 249 (43.5) | 222 (38.9) | 184 (32.1) | 259 (45.4) ^‡^ | 232 (40.5) | 242 (42.4) |
| **Smoking (***n*, %**)** | 301 (52.5) | 259 (45.4) ^‡^ | 297 (51.8) | 266 (46.6) | 275 (48.0) | 303 (53.1) | 278 (48.5) | 302 (52.9) | 294 (51.3) | 275 (48.2) | 304 (53.1) | 275 (48.2) | 318 (55.5) | 261 (45.7) ^‡^ |
| **Supplements (***n*, %**)** | 108 (18.9) | 73 (12.8) ^‡^ | 65 (11.3) | 101 (17.7) ^‡^ | 97 (16.9) | 67 (11.7) ^‡^ | 110 (19.2) | 73 (12.8) ^‡^ | 86 (15.0) | 103 (18.0) ^‡^ | 61 (10.7) | 106 (18.6) ^‡^ | 63 (11.0) | 104 (18.2) ^‡^ |
| **No dietary change (***n*, %**)** | 423 (73.8) | 449 (78.6) ^‡^ | 483 (84.3) | 411 (72.0) ^‡^ | 429 (74.9) | 454 (79.5) | 438 (76.4) | 453 (79.3) | 457 (79.8) | 420 (73.6) ^‡^ | 478 (83.4) | 393 (68.8) ^‡^ | 480 (83.8) | 411 (72.0) ^‡^ |
| **Physical activity (**MET/hours/week**)** | 124.1  (94.9, 187.6) | 139.0  (104.1, 243.9) ^†^ | 141.6  (101.2, 250.8) | 120.4  (94.9, 188.0) ^†^ | 127.4  (96.9, 196.0) | 138.1  (103.5, 250.5) ^†^ | 127.7  (96.7, 201.7) | 135.1  (101.1, 254.2) ^†^ | 140.0  (100.7, 235.3) | 124.1  (94.6, 194.0) ^†^ | 139.8  (100.3, 256.2) | 120.6  (95.6, 188.7) ^†^ | 143.4  (102.7, 250.5) | 120.7  (94.9, 189.9) ^†^ |
| **Abstinence time (**days**)** | 4.0 (3.0, 5.0) | 4.0 (3.0, 5.0) | 4.0 (3.0, 5.0) | 4.0 (3.0, 5.0) | 4.0 (3.0, 5.0) | 4.0 (3.0, 5.0) | 4.0 (3.0, 5.0) | 4.0 (3.0, 5.0) | 4.0 (3.0, 5.0) | 4.0 (3.0, 5.0) | 4.0 (3.0, 5.0) | 4.0 (3.0, 5.0) | 4.0 (3.0, 5.0) | 4.0 (3.0, 5.0) |
| **Semen parameters** |  |  |  |  |  |  |  |  |  |  |  |  |  |  |
| Ejaculate volume (mL) | 3.1 (2.4, 4.0) | 3.4 (2.5, 4.3) ^†^ | 3.2 (2.4, 4.0) | 3.4 (2.5, 4.3) | 3.0 (2.3, 4.0) | 3.5 (2.7, 4.5) ^†^ | 3.2 (2.4, 4.0) | 3.4 (2.5, 4.2) ^†^ | 3.2 (2.4, 4.0) | 3.2 (2.4, 4.2) | 3.2 (2.4, 4.0) | 3.4 (2.5, 4.4) | 3.2 (2.4, 4.2) | 3.4 (2.4, 4.3) |
| Total sperm count (10^6^/mL) | 152.6 (86.8, 255.4) | 164.9 (84.7, 264.4) | 163.2 (88.2, 260.1) | 158.5 (86.7, 263.7) | 148.7 (80.0, 248.5) | 164.9 (88.9, 260.1) ^†^ | 150.7 (82.0, 255.4) | 160.2 (86.9, 260.8) | 160.4 (89.9, 264.2) | 155.1 (82.9, 255.9) | 162.4 (86.9, 256.3) | 152.8 (86.8, 262.3) | 163.2 (88.2, 262.1) | 158.6 (89.3, 270.3) |
| Concentration (10^6^/mL) | 49.1 (28.3, 75.1) | 46.7 (28.1, 73.4) | 49.1 (28.8, 75.1) | 48.9 (28.2, 76.8) | 46.3 (27.5, 75.0) | 48.0 (27.9, 75.4) | 46.5 (28.8, 74.9) | 48.0 (28.7, 76.1) | 48.9 (28.5, 75.7) | 49.4 (28.1, 76.2) | 49.1 (28.8, 74.0) | 47.6 (27.6, 75.9) | 47.4 (28.2, 74.7) | 49.6 (30.1, 78.0) |
| Progressive motility (%) | 30.4 (19.4, 40.4) | 30.5 (17.8, 41.7) | 30.4 (18.8, 41.0) | 30.6 (19.7, 42.2) | 28.4 (18.2, 40.2) | 31.2 (19.4, 43.7) ^†^ | 28.8 (18.3, 40.4) | 31.4 (19.8, 43.7) | 30.0 (18.3, 40.5) | 29.8 (19.9, 41.4) | 29.5 (18.2, 40.5) | 29.9 (19.1, 41.4) | 30.4 (19.2, 40.4) | 29.9 (19.4, 41.2) |
| Total motility (%) | 37.0 (24.2, 49.2) | 36.7 (23.4, 53.0) | 37.1 (23.8, 51.4) | 37.3 (24.9, 52.0) | 35.9 (22.1, 48.2) | 38.1 (24.8, 53.3) ^†^ | 36.4 (21.7, 49.1) | 38.5 (24.9, 53.3) | 37.1 (22.7, 50.5) | 36.4 (25.4, 50.7) | 36.2 (22.9, 50.6) | 36.6 (23.9, 50.7) | 37.1 (24.2, 50.2) | 36.4 (24.2, 50.9) |
| Normal sperm morphology (%) | 4.0 (3.0, 6.0) | 4.0 (3.0, 7.0) | 4.0 (3.0, 7.0) | 4.0 (3.0, 7.0) | 4.0 (3.0, 7.0) | 4.0 (3.0, 6.0) | 4.0 (3.0, 7.0) | 4.0 (3.0, 7.0) | 4.0 (3.0, 6.0) | 4.0 (3.0, 7.0) | 4.0 (3.0, 6.0) | 4.0 (3.0, 7.0) | 4.0 (3.0, 7.0) | 4.0 (3.0, 7.0) |
| **Food groups (**g/d**)** |  |  |  |  |  |  |  |  |  |  |  |  |  |  |
| Staple food | 616.3 (501.9, 716.0) | 688.7 (605.3, 789.0) ^†^ | 709.6 (655.6, 792.3) | 519.5 (437.9, 611.3) ^†^ | 673.9 (550.6, 794.9) | 622.8 (502.0, 691.7) ^†^ | 632.0 (521.2, 749.9) | 650.4 (544.6, 728.0) ^†^ | 704.3 (630.0, 794.3) | 539.0 (452.5, 655.0) ^†^ | 708.8 (660.7, 776.3) | 522.6 (435.0, 614.4) ^†^ | 714.6 (637.3, 819.0) | 524.3 (440.9, 625.2) ^†^ |
| Dairy | 95.5 (46.2, 179.7) | 83.0 (33.4, 185.0) | 73.8 (38.0, 134.2) | 108.7 (62.6, 200.5) ^†^ | 87.5 (43.1, 170.5) | 90.7 (34.4, 181.9) | 86.4 (40.0, 158.8) | 90.7 (35.3, 198.4) | 80.3 (35.3, 147.7) | 104.0 (52.5, 194.0) ^†^ | 71.6 (44.8, 124.8) | 110.2 (62.1, 201.9) ^†^ | 70.7 (34.4, 130.9) | 115.0 (62.6, 215.6) ^†^ |
| Meat | 107.6 (79.4, 136.2) | 112.6 (80.8, 139.4) | 99.1 (70.5, 122.8) | 114.2 (60.0, 140.0) ^†^ | 102.2 (74.9, 127.9) | 122.1 (94.4, 150.1) ^†^ | 98.3 (72.5, 124.7) | 123.3 (95.4, 154.4) ^†^ | 95.2 (67.5, 121.2) | 120.1 (90.3, 145.7) ^†^ | 108.1 (75.6, 133.3) | 112.5 (85.9, 138.5) ^†^ | 95.6 (67.5, 121.3) | 120.2 (91.6, 146.8) ^†^ |
| Egg | 33.5 (18.8, 52.9) | 31.3 (16.4, 48.0) ^†^ | 27.7 (18.0, 44.4) | 34.9 (21.7, 55.6) ^†^ | 33.9 (18.8, 51.9) | 31.5 (18.2, 48.7) ^†^ | 32.9 (18.3, 50.8) | 32.4 (17.8, 48.1) | 30.4 (18.8, 48.0) | 34.0 (19.8, 54.3) ^†^ | 27.1 (17.8, 42.3) | 36.4 (21.5, 57.9) ^†^ | 26.8 (16.3, 42.3) | 35.6 (21.4, 57.4) ^†^ |
| Fish | 23.5 (14.4, 33.7) | 18.6 (8.9, 28.3) ^†^ | 20.1 (12.3, 29.4) | 23.9 (14.5, 33.9) ^†^ | 22.7 (13.9, 31.6) | 20.3 (10.7, 30.2) ^†^ | 23.1 (13.9, 33.0) | 19.3 (10.1, 29.4) ^†^ | 20.9 (12.3, 30.1) | 23.0 (13.3, 32.7) | 20.3 (12.9, 28.7) | 23.9 (14.2, 34.0) ^†^ | 19.0 (11.4, 28.2) | 23.8 (14.5, 34.2) ^†^ |
| Vegetable | 251.4 (187.5, 346.1) | 205.0 (155.1, 278.8) ^†^ | 231.4 (184.7, 304.3) | 257.9 (201.3, 336.2) ^†^ | 247.7 (184.6, 336.2) | 216.8 (157.3, 292.6) ^†^ | 256.9 (190.3, 346.3) | 206.9 (154.3, 286.7) ^†^ | 246.3 (188.5, 328.6) | 239. 0 (168.0, 318.0) ^†^ | 224.6 (184.6, 285.7) | 262.0 (196.4, 352.9) ^†^ | 231.5 (180.7, 312.9) | 246.1 (179.1, 329.6) ^†^ |
| Bean | 81.6 (53.6, 124.0) | 75.1 (48.0, 129.2) ^†^ | 84.4 (59.7, 123.0) | 90.9 (63.9, 132.7) ^†^ | 86.1 (55.4, 131.5) | 74.9 (48.2, 116.3) ^†^ | 92.1 (60.6, 152.8) | 67.3 (44.0, 98.3) ^†^ | 87.9 (59.6, 138.4) | 83.3 (51.4, 116.1) ^†^ | 77.1 (58.4, 100.9) | 90.9 (63.5, 141.2) ^†^ | 83.2 (55.8, 128.9) | 87.7 (58.6, 130.6) ^†^ |
| Pickled food | 17.6 (9.8, 24.7) | 13.2 (7.3, 21.7) ^†^ | 16.9 (10.3, 23.6) | 17.8 (10.9, 24.7) ^†^ | 16.1 (9.3, 23.7) | 15.6 (8.1, 24.2) ^†^ | 17.2 (9.7, 25.2) | 14.6 (7.7, 23.4) ^†^ | 16.7 (10.0, 24.5) | 17.1 (9.9, 25.1) | 17.3 (11.9, 22.5) | 17.7 (10.5, 24.5) ^†^ | 16.9 (10.2, 24.4) | 17.1 (10.4, 24.6) ^†^ |
| Fruit | 149.1 (94.1, 239.1) | 105.2 (58.1, 167.8) ^†^ | 141.2 (99.3, 210.7) | 146.8 (97.1, 214.5) ^†^ | 151.7 (97.0, 241.8) | 110.1 (59.8, 166.1) ^†^ | 163.3 (103.8, 252.7) | 104.8 (58.1, 158.8) ^†^ | 150.0 (98.2, 238.9) | 133.8 (76.9, 193.0) ^†^ | 135.9 (97.2, 185.1) | 146.1 (92.9, 221.5) ^†^ | 150.0 (99.3, 238.7) | 137.3 (79.2, 195.3) ^†^ |
| Snacks | 47.4 (29.9, 68.0) | 42.8 (26.2, 69.6) ^†^ | 47.2 (32.8, 65.1) | 54.1 (36.9, 71.8) ^†^ | 49.7 (33.2, 70.6) | 41.4 (23.8, 62.1) ^†^ | 53.9 (35.4, 78.3) | 38.7 (22.3, 56.0) ^†^ | 46.1 (28.8, 68.6) | 51.6 (33.5, 70.8) ^†^ | 47.1 (35.0, 60.9) | 53.5 (34.8, 74.1) ^†^ | 48.5 (32.7, 70.9) | 51.2 (32.5, 68.8) ^†^ |
| Tea/coffee | 317.7 (182.6, 555.0) | 8.9 (-18.5, 31.8) ^†^ | 59.3 (25.7, 194.2) | 70.7 (33.5, 236.1) ^†^ | 270.2 (89.4, 547.2) | 20.4 (-8.3, 51.9)^†^ | 259.2 (74.5, 547.2) | 21.3 (-4.8, 51.5) ^†^ | 64.5 (21.8, 262.4) | 65.6 (18.9, 193.9) ^†^ | 52.6 (28.9, 122.6) | 73.6 (31.6, 288.4) ^†^ | 55.8 (17.2, 165.6) | 70.7 (28.6, 253.6) ^†^ |
| Alcoholic beverages | 66.5 (19.2, 242.2) | 36.6 (0.1, 161.9) ^†^ | 64.8 (27.5, 253.9) | 72.2 (28.3, 240.9) ^†^ | 50.2 (12.3, 136.9) | 64.9 (9.0, 410.3)^†^ | 51.9 (12.9, 206.2) | 55.5 (9.0, 295.6) | 67.6 (23.7, 318.0) | 57.5 (12.3, 154.8) ^†^ | 60.1 (28.6, 118.9) | 73.1 (26.8, 257.1) ^†^ | 61.1 (18.7, 242.7) | 63.1 (17.3, 217.9) |
| Nonalcoholic beverage | 50.8 (21.6, 114.4) | 39.5 (11.4, 120.0) ^†^ | 50.0 (24.8, 114.4) | 53.2 (27.8, 114.8) ^†^ | 47.6 (21.1, 109.0) | 49.6 (17.2, 130.5) | 48.3 (22.4, 113.4) | 44.9 (16.8, 126.3) | 44.5 (18.5, 97.7) | 55.0 (27.6, 124.4) ^†^ | 47.9 (27.2, 82.9) | 53.1 (25.2, 116.9) ^†^ | 69.5 (29.4, 157.9) | 43.9 (20.3, 81.2) ^†^ |
| **Nutrients** |  |  |  |  |  |  |  |  |  |  |  |  |  |  |
| Energy (kcal/d) | 1728.2 (1429.5, 2144.1) | 1900.0 (1600.0, 2300.0) ^†^ | 1588.0 (1348.4, 1948.3) | 1586.9 (1261.7, 1974.9) ^†^ | 1735.9 (1427.4, 2154.6) | 1800.8 (1520.9, 2266.5) ^†^ | 1747.8 (1426.4, 2175.1) | 1782.6 (1527.4, 2226.5) ^†^ | 1701.2 (1402.2, 2138.7) | 1720.0 (1340.0, 2130.0) | 1504.5 (1314.5, 1650.6) | 1658.9 (1355.9, 2027.9) ^†^ | 1699.5 (1419.1, 2128.0) | 1684.9 (1330.2, 2115.2) |
| Carbohydrate (%E) | 56.2 (51.5, 60.8) | 57.0 (53.0, 62.0) ^†^ | 60.6 (56.4, 64.8) | 53.2 (49.1, 57.7) ^†^ | 58.2 (53.4, 62.9) | 54.7 (49.9, 58.3) ^†^ | 57.7 (53.0, 62.0) | 55.7 (50.9, 59.8) ^†^ | 59.8 (54.6, 64.4) | 54.0 (50.0, 58.0) ^†^ | 60.9 (56.9, 65.3) | 53.0 (48.5, 57.5) ^†^ | 61.0 (56.7, 65.4) | 52.8 (48.5, 56.8) ^†^ |
| Fat (%E) | 25.2 (22.4, 28.2) | 25.0 (22.0 28.0) ^†^ | 22.2 (19.2, 24.4) | 27.6 (24.7, 30.7) ^†^ | 24.8 (21.8, 27.7) | 25.8 (23.2, 29.0) ^†^ | 25.1 (22.2, 28.0) | 25.6 (22.9, 28.5) ^†^ | 22.7 (19.5, 25.2) | 28.0 (25.0, 31.0) ^†^ | 22.3 (19.2, 25.0) | 27.6 (24.7, 30.7) ^†^ | 22.1 (19.2, 24.7) | 28.2 (25.3, 30.9) ^†^ |
| Protein (%E) | 16.6 (15.4, 18.0) | 17.0 (15.0, 18.0) ^†^ | 15.8 (14.6, 17.1) | 17.1 (15.9, 18.7) ^†^ | 16.4 (15.1, 17.7) | 16.9 (15.5, 18.2) ^†^ | 16.4 (15.1, 17.7) | 16.8 (15.4, 17.9) ^†^ | 16.0 (14.8, 17.3) | 17.0 (16.0, 18.0) ^†^ | 15.9 (14.6, 17.1) | 17.1 (15.9, 18.7) ^†^ | 15.6 (14.3, 16.7) | 17.3 (16.2, 18.8) ^†^ |
| Fiber (g/d) | 17.9 (15.0, 22.0) | 15.5 (13.3, 18.3) ^†^ | 16.9 (14.8, 19.6) | 18.6 (15.7, 22.1) ^†^ | 17.7 (14.9, 22.0) | 15.6 (13.3, 18.6) ^†^ | 18.5 (15.4, 23.1) | 15.2 (13.0, 17.7) ^†^ | 17.6 (15.2, 20.9) | 17.5 (14.1, 20.6) ^†^ | 16.4 (14.6, 18.2) | 18.7 (15.9, 22.5) ^†^ | 17.2 (14.7, 20.2) | 17.9 (14.8, 21.2) ^†^ |
| Vitamin A (ugRE) | 584.3  (410.7, 814.9) | 485.4  (319.7, 715.9) ^†^ | 517.5  (400.9, 744.6) | 632.8  (464.1, 865.4) ^†^ | 561.6  (391.8, 766.3) | 544.8  (348.0, 788.0) | 569.7  (403.1, 787.8) | 527.5  (345.5, 773.8) | 530.4  (398.3, 737.2) | 599.0  (413.0, 827.0) ^†^ | 509.4  (403.1, 727.9) | 628.8  (450.0, 848.2) ^†^ | 495.1  (368.1, 701.7) | 623.0  (445.8, 848.2) ^†^ |
| Vitamin C (mg/d) | 116.1 (88.1, 151.1) | 84.4 (64.5, 110.0) ^†^ | 108.8 (86.3, 136.6) | 112.9 (89.1, 145.1) ^†^ | 110.1 (83.6, 145.4) | 98.7 (71.8, 131.2) ^†^ | 115.3 (87.8, 152.7) | 91.2 (67.2, 117.4) ^†^ | 116.7 (91.5, 153.8) | 103.0 (71.9, 131.0) ^†^ | 96.1 (79.6, 118.0,) | 116.3 (90.3, 156.3) ^†^ | 110.9 (85.5, 143.9) | 107.3 (78.3, 137.3) ^†^ |
| Vitamin E (mg/d) | 15.7 (12.4, 19.7) | 14.2 (11.2, 19.2) ^†^ | 15.0 (12.6, 18.3) | 17.3 (14.4, 21.2) ^†^ | 15.8 (12.7, 20.2) | 14.5 (11.3, 18.3) ^†^ | 17.0 (13.4, 22.0) | 13.4 (10.5, 16.8) ^†^ | 15.4 (12.8, 19.6) | 16.4 (12.3, 19.7) ^†^ | 14.4 (12.4, 16.9) | 17.2 (14.3, 22.1) ^†^ | 14.9 (12.4, 18.8) | 16.9 (13.3, 20.8) ^†^ |
| Carotenoid (ug/d) | 2388.8 (1554.6, 3300.0) | 1769.1 (1212.2, 2569.7) ^†^ | 2143.8 (1503.7, 2842.3) | 2507.0 (1771.0, 3303.3) ^†^ | 2376.5 (1522.7, 3349.2) | 1855.2 (1255.9, 2716.1) ^†^ | 2507.8 (1636.6, 3557.6) | 1774.0 (1218.3, 2575.7) ^†^ | 2301.0 (1562.1, 3273.9) | 2305.0 (1380.0, 3103.0) ^†^ | 2072.5 (1554.6, 2650.6) | 2514.3 (1774.0, 3662.9) ^†^ | 2137.7 (1470.8, 2924.2) | 2403.6 (1568.6, 3227.3) ^†^ |
| Flavonoids (mg/d) | 88.1 (63.9, 116.8) | 71.5 (50.8, 93.5) ^†^ | 82.7 (64.9, 106.4) | 88.8 (67.4, 117.5) ^†^ | 88.8 (64.7, 117.8) | 71.4 (51.1, 94.1) ^†^ | 92.4 (68.8, 127.1) | 66.1 (48.3, 85.4) ^†^ | 88.8 (66.5, 118.2) | 81.2 (55.1, 107.0) ^†^ | 78.7 (63.5, 94.8) | 89.3 (67.2, 121.7) ^†^ | 86.1 (64.1, 115.6) | 83.0 (59.4, 111.5) ^†^ |
| Magnesium (mg/d) | 324.0 (296.5, 355.6) | 289.0 (266.0, 320.0) ^†^ | 312.3 (292.4, 335.2) | 313.7 (281.9, 344.3) ^†^ | 322.9 (295.5, 353.7) | 291.2 (267.0, 320.4) ^†^ | 327.9 (299.4, 361.8) | 286.4 (264.2, 312.3) ^†^ | 320.5 (299.0, 350.0) | 302.0 (268.0, 332.0) ^†^ | 305.0 (286.4, 323.2) | 319.0 (285.8, 353.1) ^†^ | 311.6 (290.7, 338.7) | 309.7 (275.0, 340.7) ^†^ |
| Zinc (mg/d) | 11.6 (10.6, 12.6) | 11.7 (10.6, 12.6) | 12.1 (11.4, 12.8) | 10.7 (9.8, 11.6) ^†^ | 11.5 (10.6, 12.4) | 11.8 (10.6, 12.8) | 11.4 (10.5, 12.4) | 11.7 (10.6, 12.7) ^†^ | 12.0 (11.2, 12.9) | 10.8 (9.9, 11.7) ^†^ | 12.1 (11.4, 12.8) | 10.8 (9.9, 11.6) ^†^ | 12.0 (11.2, 12.7) | 10.9 (9.9, 11.8) ^†^ |
| Selenium (ug/d) | 42.7 (37.0, 49.7) | 39.6 (34.7, 45.5) ^†^ | 38.7 (34.6, 43.3) | 46.0 (41.4, 51.6) ^†^ | 40.8 (35.4, 47.3) | 42.6 (37.2, 50.0) ^†^ | 41.8 (35.7, 48.6) | 41.5 (36.4, 48.6) | 39.2 (35.0, 44.7) | 44.8 (39.4, 51.3) ^†^ | 38.8 (35.1, 43.3) | 46.1 (40.9, 51.6) ^†^ | 38.3 (34.0, 42.8) | 45.7 (40.9, 52.1) ^†^ |
| **Cooking methods (**times/week**)** | |  |  |  |  |  |  |  |  |  |  |  |  |  |
| Deep-frying | 1.2 (0.6, 2.0) | 1.2 (0.6, 1.8) | 1.0 (0.6, 1.6) | 1.2 (0.6, 1.8) ^†^ | 1.2 (0.6, 1.8) | 1.2 (0.6, 2.0) ^†^ | 1.2 (0.6, 1.8) | 1.2 (0.6, 1.8) ^†^ | 1.0 (0.6, 1.8) | 1.2 (0.6, 1.8) ^†^ | 0.6 (0.6, 1.2) | 1.2 (0.6, 1.8) ^†^ | 1.2 (0.6, 1.8) | 1.2 (0.6, 2.0) |
| Stewing | 5.6 (3.7, 8.1) | 5.6 (3.7, 8.2) ^†^ | 5.6 (3.1, 8.1) | 5.6 (3.5, 8.1) | 5.6 (3.7, 8.1) | 5.6 (3.5, 8.4) ^†^ | 5.6 (3.7, 8.1) | 5.6 (3.5, 8.2) ^†^ | 5.6 (3.5, 8.1) | 5.6 (3.5, 8.2) | 5.0 (2.6, 7.6) | 5.6 (3.5, 8.1) ^†^ | 5.6 (3.1, 8.1) | 5.6 (3.5, 8.2) |
| Broiling | 1.2 (0.6, 2.2) | 1.2 (0.6, 1.8) ^†^ | 1.2 (0.6, 1.8) | 1.2 (0.6, 1.8) ^†^ | 1.2 (0.6, 1.8) | 1.2 (0.6, 2.2) ^†^ | 1.2 (0.6, 1.8) | 1.2 (0.6, 1.8) ^†^ | 1.2 (0.6, 1.8) | 1.2 (0.6, 2.0) ^†^ | 1.2 (0.6, 1.6) | 1.2 (0.6, 2.0) ^†^ | 1.2 (0.6, 1.8) | 1.2 (0.6, 2.0) ^†^ |
| Stir-frying | 10.0 (7.5, 12.0) | 10.0 (7.5, 12.0) ^†^ | 9.5 (7.5, 12.0) | 9.5 (7.5, 12.0) ^†^ | 10.0 (7.5, 12.0) | 10.0 (7.5, 12.0) ^†^ | 10.0 (7.5, 12.0) | 10.0 (7.5, 14.0) ^†^ | 9.5 (7.5, 12.0) | 10.0 (7.6, 12.0) ^†^ | 9.5 (7.5, 12.0) | 9.5 (7.5, 12.0) ^†^ | 9.5 (7.5, 12.0) | 10.0 (7.5, 12.0) ^†^ |
| Steaming | 0.6 (0.0, 1.2) | 0.6 (0.0, 1.2) ^†^ | 0.6 (0.0, 1.2) | 0.6 (0.0, 1.2) ^†^ | 0.6 (0.0, 1.2) | 0.6 (0.0, 1.2) ^†^ | 0.6 (0.0, 1.2) | 0.6 (0.0, 1.2) ^†^ | 0.6 (0.0, 1.2) | 0.6 (0.0, 1.2) | 0.6 (0.0, 1.0) | 0.6 (0.0, 1.2) ^†^ | 0.6 (0.0, 1.2) | 0.6 (0.0, 1.2) ^†^ |
| Raw | 1.0 (0.6, 2.5) | 1.0 (0.6, 2.5) ^†^ | 1.0 (0.6, 2.5) | 1.0 (0.6, 2.5) | 1.0 (0.6, 2.5) | 1.0 (0.6, 2.5) | 1.0 (0.6, 2.5) | 1.0 (0.0, 2.5) ^†^ | 1.0 (0.6, 2.5) | 1.0 (0.6, 2.5) ^†^ | 0.6 (0.0, 2.5) | 1.0 (0.6, 2.5) ^†^ | 1.0 (0.6, 2.5) | 1.0 (0.6, 2.5) |

FRAP: Ferric-reducing ability of plasma; GAE: Gallic acid equivalents; H-ORAC: Hydrophilic oxygen radical absorbance capacity; L-ORAC: Lipophilic oxygen radical absorbance capacity; MET: Metabolic equivalent task; No.: Number; T-ORAC: Total oxygen radical absorbance capacity; TE: Trolox equivalents; TEAC: Trolox equivalent antioxidant capacity; TP: Total phenolics; TRAP: Total radical-trapping antioxidant parameter.

Data is presented as a median (*P*_25_, *P*_75_) or as a count (%).

† *P*<0.05 (Data were analyzed by Kruskal–Wallis test).

‡ *P*<0.05 (Data were analyzed by Chi-square test).

The residual method was used to adjust the intake variables for energy intake.

**Supplementary Table S3.** Subgroup analyses of dietary total antioxidant capacity indices and semen parameters.

|  | **Ejaculate volume (**mL**)** | **Total sperm count**  **(**10^6^/mL**)** | **Sperm concentration**  **(**10^6^/mL**)** | **Progressive motility (**%**)** | **Total motility**  **(**%**)** | **Normal sperm morphology (**%**)** |
| --- | --- | --- | --- | --- | --- | --- |
| **FRAP (**mmol/d**)** | -0.015 (-0.023, -0.006) | -0.024 (-0.742, 0.695) | 0.079 (-0.212, 0.369) | -0.031 (-0.149, 0.086) | -0.038 (-0.201, 0.125) | -0.002 (-0.016, 0.012) |
| **Age (**years**)** |  |  |  |  |  |  |
| <32 | -0.007 (-0.025, 0.010) | -0.853 (-1.989, 0.282) | 0.008 (-0.415, 0.432) | -0.002 (-0.201, 0.198) | -0.007 (-0.265, 0.252) | -0.008 (-0.038, 0.023) |
| ≥32 | -0.014 (-0.025, -0.002) | 0.320 (-0.758, 1.398) | 0.290 (-0.145, 0.725) | -0.083 (-0.256, 0.091) | -0.073 (-0.288, 0.141) | 0.000 (-0.021, 0.021) |
| **Smoking** |  |  |  |  |  |  |
| Yes | -0.015 (-0.027, -0.003) | -0.139 (-1.020, 0.743) | -0.102 (-0.392, 0.189) | 0.036 (-0.113, 0.185) | 0.022 (-0.168, 0.212) | -0.001 (-0.021, 0.019) |
| No | -0.008 (-0.021, 0.005) | 0.599 (-0.542, 1.739) | 0.305 (-0.058, 0.669) | -0.062 (-0.273, 0.148) | -0.154 (-0.419, 0.112) | -0.008 (-0.039, 0.023) |
| **Nutritional Supplements use** | |  |  |  |  |  |
| Yes | -0.017 (-0.041, 0.008) | -0.780 (-2.838, 1.279) | 0.172 (-0.377, 0.721) | -0.108 (-0.346, 0.130) | -0.135 (-0.425, 0.154) | -0.010 (-0.050, 0.031) |
| No | -0.013 (-0.023, -0.004) | 0.090 (-0.779, 0.959) | 0.078 (-0.274, 0.429) | -0.020 (-0.168, 0.129) | -0.046 (-0.240, 0.148) | 0.033 (-0.016, 0.023) |
| **No dietary change** |  |  |  |  |  |  |
| Yes | -0.014 (-0.024, -0.003) | -0.308 (-1.080, 0.465) | 0.080 (-0.236, 0.397) | -0.009 (-0.165, 0.147) | 0.019 (-0.187, 0.225) | -0.004 (-0.018, 0.010) |
| No | -0.015 (-0.034, 0.005) | -0.033 (-1.879, 1.814) | 0.278 (-0.319, 0.875) | 0.019 (-0.235, 0.272) | -0.034 (-0.360, 0.291) | 0.032 (-0.019, 0.084) |
|  |  |  |  |  |  |  |
| **T-ORAC (**μmol TE/d**)** | -0.000 (-0.000, 0.000) | -0.000 (-0.000, 0.000) | -0.000 (-0.000, 0.000) | 0.000 (-0.000, 0.000) | 0.000 (-0.000, 0.000) | -0.000 (-0.000, 0.000) |
| **Age (**years**)** |  |  |  |  |  |  |
| <32 | -0.000 (-0.000, 0.000) | -0.000 (-0.000, 0.000) | -0.000 (-0.000, 0.000) | 0.000 (-0.000, 0.000) | 0.000 (-0.000, 0.000) | -0.000 (-0.000, 0.000) |
| ≥32 | -0.000 (-0.000, 0.000) | -0.000 (-0.000, 0.000) | 0.000 (-0.000, 0.000) | 0.000 (-0.000, 0.000) | -0.000 (-0.000, 0.000) | -0.000 (-0.000, 0.000) |
| **Smoking** |  |  |  |  |  |  |
| Yes | -0.000 (-0.000, 0.000) | 0.000 (-0.000, 0.000) | 0.000 (-0.000, 0.000) | 0.000 (-0.000, 0.000) | 0.000 (-0.000, 0.000) | 0.000 (-0.000, 0.000) |
| No | -0.000 (-0.000, 0.000) | -0.000 (-0.000, 0.000) | -0.000 (-0.000, 0.000) | -0.000 (-0.000, 0.000) | -0.000 (-0.000, 0.000) | -0.000 (-0.000, 0.000) |
| **Nutritional Supplements use** | |  |  |  |  |  |
| Yes | -0.000 (-0.000, 0.000) | -0.000 (-0.001, 0.001) | -0.000 (-0.000, 0.000) | -0.000 (-0.000, 0.000) | -0.000 (-0.000, 0.000) | 0.000 (-0.000, 0.000) |
| No | -0.000 (-0.000, 0.000) | 0.000 (-0.000, 0.000) | -0.000 (-0.000, 0.000) | 0.000 (-0.000, 0.000) | 0.000 (-0.000, 0.000) | -0.000 (-0.000, 0.000) |
| **No dietary change** |  |  |  |  |  |  |
| Yes | -0.000 (-0.000, 0.000) | -0.000 (-0.000, 0.000) | -0.000 (-0.000, 0.000) | -0.000 (-0.000, 0.000) | -0.000 (-0.000, 0.000) | -0.000 (-0.000, 0.000) |
| No | 0.000 (-0.000, 0.000) | 0.000 (-0.000, 0.000) | 0.000 (-0.000, 0.000) | 0.000 (-0.000, 0.000) | 0.000 (-0.000, 0.000) | -0.000 (-0.000, 0.000) |
|  |  |  |  |  |  |  |
| **TRAP (**mmol TE/d**)** | -0.019 (-0.041, 0.002) | 0.588 (-1.273, 2.450) | 0.271 (-0.512, 1.055) | -0.181 (-0.506, 0.145) | -0.137 (-0.578, 0.303) | 0.011 (-0.032, 0.053) |
| **Age (**years**)** |  |  |  |  |  |  |
| <32 | -0.016 (-0.054, 0.021) | -2.183 (-6.310, 1.944) | 0.018 (-1.268, 1.034) | -0.053 (-0.695, 0.589) | -0.153 (-0.949, 0.644) | -0.013 (-0.106, 0.081) |
| ≥32 | -0.021 (-0.053, 0.011) | 0.796 (-1.868, 3.459) | 0.762 (-0.191, 1.714) | -0.162 (-0.575, 0.251) | -0.151 (-0.673, 0.372) | 0.010 (-0.044, 0.064) |
| **Smoking** |  |  |  |  |  |  |
| Yes | -0.020 (-0.050, 0.010) | 0.243 (-2.745, 3.231) | -0.188 (-1.184, 0.808) | 0.101 (-0.286, 0.487) | 0.252 (-0.270, 0.775) | 0.018 (-0.043, 0.079) |
| No | -0.010 (-0.042, 0.022) | 0.921 (-1.773, 3.614) | 0.848 (-0.045, 1.741) | -0.402 (-0.784, -0.019) | -0.389 (-0.817, 0.040) | 0.002 (-0.068, 0.072) |
| **Nutritional Supplements use** | |  |  |  |  |  |
| Yes | -0.035 (-0.093, 0.024) | -0.814 (-5.546, 3.918) | 1.160 (-0.025, 2.344) | 0.141 (-0.681, 0.963) | 0.084 (-0.878, 1.046) | -0.027 (-0.130, 0.076) |
| No | -0.020 (-0.046, 0.006) | 0.708 (-1.566, 2.982) | 0.141 (-0.723, 1.005) | -0.211 (-0.535, 0.113) | -0.341 (-0.789, 0.107) | 0.015 (-0.035, 0.065) |
| **No dietary change** |  |  |  |  |  |  |
| Yes | -0.017 (-0.043, 0.010) | -0.189 (-2.166, 1.787) | -0.100 (-0.900, 0.700) | -0.103 (-0.459, 0.254) | -0.048 (-0.561, 0.465) | -0.008 (-0.046, 0.029) |
| No | -0.033 (-0.077, 0.011) | -0.128 (-5.091, 4.835) | 0.936 (-0.334, 2.206) | 0.079 (-0.638, 0.796) | -0.046 (-0.897, 0.806) | 0.123 (0.013, 0.232) |
|  |  |  |  |  |  |  |
| **TEAC (**mmol TE/d**)** | -0.034 (-0.068, -0.000) | 1.047(-1.714, 3.807) | 0.514 (-0.625, 1.652) | -0.306 (-0.787, 0.174) | -0.243 (-0.905, 0.419) | 0.015 (-0.047, 0.077) |
| **Age (**years**)** |  |  |  |  |  |  |
| <32 | -0.031 (-0.085, 0.024) | -2.798 (-8.722, 3.125) | 0.394 (-1.544, 2.331) | -0.046 (-0.968, 0.877) | -0.035 (-1.151, 1.081) | 0.037 (-0.100, 0.174) |
| ≥32 | -0.036 (-0.083, 0.011) | 1.130 (-2.608, 4.868) | 0.568 (-0.810, 1.946) | -0.210 (-0.818, 0.399) | -0.309 (-1.070, 0.453) | 0.009 (-0.069, 0.086) |
| **Smoking** |  |  |  |  |  |  |
| Yes | -0.036 (-0.081, 0.009) | 0.260 (-3.737, 4.256) | -0.327 (-1.738, 1.085) | 0.175 (-0.417, 0.767) | 0.390 (-0.403, 1.183) | 0.029 (-0.057, 0.115) |
| No | -0.019 (-0.066, 0.028) | 1.723 (-2.160, 5.606) | 1.243 (-0.010, 2.496) | -0.579 (-1.168, 0.011) | -0.608 (-1.301, 0.086) | 0.003 (-0.098, 0.104) |
| **Nutritional Supplements use** | |  |  |  |  |  |
| Yes | -0.074 (-0.164, 0.017) | -1.540 (-9.043, 5.974) | 1.680 (-0.248, 3.608) | -0.071 (-1.272, 1.289) | 0.059 (-1.317, 1.436) | -0.031 (-0.194, 0.132) |
| No | -0.035 (-0.075, 0.006) | 1.199 (-1.996, 4.394) | 0.390 (-0.940, 1.720) | -0.334 (-0.852, 0.185) | -0.427 (-1.123, 0.269) | 0.223 (-0.053, 0.099) |
| **No dietary change** |  |  |  |  |  |  |
| Yes | -0.027 (-0.065, 0.011) | -0.263 (-3.031, 2.505) | 0.013 (-1.151, 1.176) | -0.206 (-0.724, 0.311) | -0.027 (-0.754, 0.700) | -0.014 (-0.066, 0.039) |
| No | -0.066 (-0.131, -0.001) | -0.182 (-7.811, 7.447) | 1.781 (-0.107, 3.669) | 0.120 (-0.869, 1.109) | -0.096 (-1.247, 1.055) | 0.182 (0.017, 0.347) |
|  |  |  |  |  |  |  |
| **H-ORAC**  **(**μmol TE/d**)** | -0.000 (-0.000, 0.000) | -0.000 (-0.000, 0.000) | -0.000 (-0.000, 0.000) | 0.000 (-0.000, 0.000) | 0.000 (-0.000, 0.000) | -0.000 (-0.000, 0.000) |
| **Age (**years**)** |  |  |  |  |  |  |
| <32 | -0.000 (-0.000, 0.000) | -0.000 (-0.001, 0.001) | -0.000 (-0.000, 0.000) | -0.000 (-0.000, 0.000) | 0.000 (-0.000, 0.000) | -0.000 (-0.000, 0.000) |
| ≥32 | -0.000 (-0.000, 0.000) | -0.000 (-0.001, 0.001) | -0.000 (-0.000, 0.000) | 0.000 (-0.000, 0.000) | -0.000 (-0.000, 0.000) | -0.000 (-0.000, 0.000) |
| **Smoking** |  |  |  |  |  |  |
| Yes | -0.000 (-0.000, 0.000) | 0.000 (-0.001, 0.001) | 0.000 (-0.000, 0.000) | 0.000 (-0.000, 0.000) | 0.000 (-0.000, 0.000) | 0.000 (-0.000, 0.000) |
| No | -0.000 (-0.000, 0.000) | -0.000 (-0.001, 0.001) | -0.000 (-0.000, 0.000) | -0.000 (-0.000, 0.000) | -0.000 (-0.000, 0.000) | -0.000 (-0.000, 0.000) |
| **Nutritional Supplements use** | |  |  |  |  |  |
| Yes | -0.000 (-0.000, 0.000) | -0.000 (-0.001, 0.001) | -0.000 (-0.000, 0.000) | -0.000 (-0.000, 0.000) | -0.000 (-0.000, 0.000) | 0.000 (-0.000, 0.000) |
| No | -0.000 (-0.000, 0.000) | -0.000 (-0.001, 0.000) | -0.000 (-0.000, 0.000) | 0.000 (-0.000, 0.000) | 0.000 (-0.000, 0.000) | -0.000 (-0.000, 0.000) |
| **No dietary change** |  |  |  |  |  |  |
| Yes | -0.000 (-0.000, 0.000) | -0.000 (-0.001, 0.000) | -0.000 (-0.000, 0.000) | -0.000 (-0.000, 0.000) | -0.000 (-0.000, 0.000) | -0.000 (-0.000, 0.000) |
| No | 0.000 (-0.000, 0.000) | 0.001 (-0.000, 0.002) | 0.000 (-0.000, 0.000) | 0.000 (-0.000, 0.000) | 0.000 (-0.000, 0.000) | -0.000 (-0.000, 0.000) |
|  |  |  |  |  |  |  |
| **L-ORAC (**μmol TE/d**)** | -0.000 (-0.000, 0.000) | 0.000 (-0.000, 0.000) | -0.000 (-0.000, 0.000) | 0.000 (-0.000, 0.000) | 0.000 (-0.000, 0.000) | -0.000 (-0.000, 0.000) |
| **Age (**years**)** |  |  |  |  |  |  |
| <32 | -0.000 (-0.000, 0.000) | -0.000 (-0.001, 0.001) | -0.000 (-0.000, 0.000) | 0.000 (-0.000, 0.000) | 0.000 (-0.000, 0.000) | 0.000 (-0.000, 0.000) |
| ≥32 | 0.000 (-0.000, 0.000) | 0.000 (-0.000, 0.001) | 0.000 (-0.000, 0.000) | 0.000 (-0.000, 0.000) | -0.000 (-0.000, 0.000) | -0.000 (-0.000, 0.000) |
| **Smoking** |  |  |  |  |  |  |
| Yes | -0.000 (-0.000, 0.000) | 0.000 (-0.000, 0.001) | 0.000 (-0.000, 0.000) | 0.000 (-0.000, 0.000) | 0.000 (-0.000, 0.000) | 0.000 (-0.000, 0.000) |
| No | -0.000 (-0.000, 0.000) | 0.000 (-0.000, 0.001) | -0.000 (-0.000, 0.000) | -0.000 (-0.000, 0.000) | -0.000 (-0.000, 0.000) | -0.000 (-0.000, 0.000) |
| **Nutritional Supplements use** | |  |  |  |  |  |
| Yes | -0.000 (-0.000, 0.000) | -0.000 (-0.001, 0.001) | 0.000 (-0.000, 0.000) | 0.000 (-0.000, 0.000) | 0.000 (-0.000, 0.000) | 0.000 (-0.000, 0.000) |
| No | -0.000 (-0.000, 0.000) | 0.000 (-0.000, 0.000) | -0.000 (-0.000, 0.000) | 0.000 (-0.000, 0.000) | -0.000 (-0.000, 0.000) | -0.000 (-0.000, 0.000) |
| **No dietary change** |  |  |  |  |  |  |
| Yes | -0.000 (-0.000, 0.000) | -0.000 (-0.000, 0.000) | -0.000 (-0.000, 0.000) | -0.000 (-0.000, 0.000) | 0.000 (-0.000, 0.000) | -0.000 (-0.000, 0.000) |
| No | -0.000 (-0.000, 0.000) | 0.000 (-0.001, 0.001) | -0.000 (-0.000, 0.000) | 0.000 (-0.000, 0.000) | 0.000 (-0.000, 0.000) | -0.000 (-0.000, 0.000) |
|  |  |  |  |  |  |  |
| **TP (**mg GAE/d**)** | -0.000 (-0.000, 0.000) | -0.004 (-0.011, 0.004) | -0.002 (-0.004, 0.000) | 0.000 (-0.001, 0.001) | -0.000 (-0.001, 0.001) | -0.000 (-0.000, 0.000) |
| **Age (**years**)** |  |  |  |  |  |  |
| <32 | -0.000 (-0.000, 0.000) | -0.005 (-0.018, 0.009) | -0.001 (-0.005, 0.003) | 0.000 (-0.002, 0.002) | 0.000 (-0.002, 0.002) | 0.000 (-0.000, 0.000) |
| ≥32 | -0.000 (-0.000, 0.000) | -0.004 (-0.015, 0.007) | -0.002 (-0.005, 0.002) | 0.000 (-0.001, 0.002) | -0.000 (-0.002, 0.002) | -0.000 (-0.000, 0.000) |
| **Smoking** |  |  |  |  |  |  |
| Yes | -0.000 (-0.000, 0.000) | -0.000 (-0.011, 0.010) | -0.001 (-0.004, 0.002) | -0.000 (-0.002, 0.001) | -0.000 (-0.002, 0.001) | 0.000 (-0.000, 0.000) |
| No | -0.000 (-0.000, 0.000) | -0.004 (-0.017, 0.008) | -0.002 (-0.005, 0.002) | 0.000 (-0.002, 0.002) | -0.000 (-0.002, 0.002) | -0.000 (-0.000, 0.000) |
| **Nutritional Supplements use** | |  |  |  |  |  |
| Yes | -0.000 (-0.000, 0.000) | -0.011 (-0.034, 0.013) | -0.002 (-0.009, 0.005) | -0.001 (-0.005, 0.002) | -0.001 (-0.005, 0.003) | 0.000 (-0.000, 0.001) |
| No | -0.000 (-0.000, 0.000) | -0.002 (-0.011, 0.007) | -0.002 (-0.005, 0.001) | 0.000 (-0.001, 0.002) | -0.000 (-0.002, 0.001) | -0.000 (-0.000, 0.000) |
| **No dietary change** |  |  |  |  |  |  |
| Yes | -0.000 (-0.000, 0.000) | -0.006 (-0.014, 0.003) | -0.002 (-0.005, 0.000) | -0.000 (-0.001, 0.001) | -0.000 (-0.002, 0.002) | -0.000 (-0.000, 0.000) |
| No | -0.000 (-0.000, 0.000) | 0.006 (-0.013, 0.025) | -0.000 (-0.006, 0.005) | 0.002 (-0.001, 0.004) | 0.001 (-0.002, 0.004) | 0.000 (-0.000, 0.001) |

FRAP: Ferric-reducing ability of plasma; GAE: Gallic acid equivalents; H-ORAC: Hydrophilic oxygen radical absorbance capacity; L-ORAC: Lipophilic oxygen radical absorbance capacity; T-ORAC: Total oxygen radical absorbance capacity; TE: Trolox equivalents; TEAC: Trolox equivalent antioxidant capacity; TP: Total phenolics; TRAP: Total radical-trapping antioxidant parameter.

The model was adjusted for total energy intake (kcal/day), region (Liaoning/other provinces), age (years), BMI (kg/m^2^), fiber intake (g/day), annual family income (RMB; thousand yuan), physical activity (MET/hours/week), abstinence time (days), smoking (yes/no), drinking (yes/no), education (junior secondary or below, senior high school/technical secondary school, and junior college/university or above), occupation (employed/unemployed), nutritional supplements use (yes/no), dietary change (yes/no), and cooking methods (times/week).

**Supplementary Table S4.** The results of the additive and multiplicative interaction analyses.

|  | | ***P* for multiplicative interaction** | **RERI for additive interaction** |
| --- | --- | --- | --- |
| **Ejaculate volume** | |  |  |
| **FRAP** | Age | 0.049 | 0.712 (-0.186, 1.611) |
|  | Smoking | 0.539 | 0.100 (-0.683, 0.884) |
|  | Nutritional supplements use | 0.564 | 0.123 (-0.686, 0.932) |
|  | No dietary change | 0.025 | -1.616 (-3.053, -0.179) |
| **T-ORAC** | Age | 0.327 | -0.317 (-1.198, 0.564) |
|  | Smoking | 0.400 | 0.152 (-0.582, 0.885) |
|  | Nutritional supplements use | 0.584 | -0.323 (-1.126, 0.480) |
|  | No dietary change | 0.142 | 0.648 (-0.386, 1.683) |
| **TRAP** | Age | 0.382 | 0.299 (-0.561, 1.160) |
|  | Smoking | 0.105 | 0.436 (-0.339, 1.212) |
|  | Nutritional supplements use | 0.067 | 0.644 (-0.134, 1.421) |
|  | No dietary change | 0.049 | -1.320 (-2.610, -0.029) |
| **TEAC** | Age | 0.669 | 0.170 (-0.706, 1.046) |
|  | Smoking | 0.743 | -0.010 (-0.792, 0.772) |
|  | Nutritional supplements use | 0.606 | 0.084 (-0.716, 0.885) |
|  | No dietary change | 0.063 | -1.246 (-2.528, 0.036) |
| **H-ORAC** | Age | 0.343 | -0.302 (-1.176, 0.572) |
|  | Smoking | 0.985 | -0.123 (-0.880, 0.634) |
|  | Nutritional supplements use | 0.995 | -0.127 (-0.895, 0.641) |
|  | No dietary change | 0.570 | -0.396 (-1.457, 0.665) |
| **L-ORAC** | Age | 0.745 | 0379 (-0.494, 1.252) |
|  | Smoking | 0.571 | 0.183 (-0.574, 0.939) |
|  | Nutritional supplements use | 0.384 | -0.334 (-1.241, 0.574) |
|  | No dietary change | 0.692 | 0.215 (-0.806, 1.235) |
| **TP** | Age | 0.283 | 0.006 (-0.886, 0.897) |
|  | Smoking | 0.572 | 0.244 (-0.527, 1.015) |
|  | Nutritional supplements use | 0.752 | 0.173 (-0.624, 0.969) |
|  | No dietary change | 0.398 | 0.491 (-0.541, 1.523) |
| **Total sperm count** | |  |  |
| **FRAP** | Age | 0.132 | 0.304 (-0.218, 0.827) |
|  | Smoking | 0.954 | 0.027 (-0.534, 0.588) |
|  | Nutritional supplements use | 0.858 | -0093 (-0.768, 0.581) |
|  | No dietary change | 0.981 | -0.002 (-0769, 0.765) |
| **T-ORAC** | Age | 0.942 | 0.002 (-0.551, 0.556) |
|  | Smoking | 0.455 | 0.337 (-0.240, 0.914) |
|  | Nutritional supplements use | 0.271 | -0.279 (-1.007, 0.449) |
|  | No dietary change | 0.612 | 0.264 (-0.493, 1.021) |
| **TRAP** | Age | 0.725 | -0.088 (-0.601, 0.426) |
|  | Smoking | 0.869 | -0.067 (-0.610, 0.476) |
|  | Nutritional supplements use | 0.537 | 0.014 (-0.543, 0.571) |
|  | No dietary change | 0.464 | 0.141 (-0.614, 0.896) |
| **TEAC** | Age | 0.679 | -0.219 (-0.751, 0.314) |
|  | Smoking | 0.926 | -0.062 (-0.614, 0.491) |
|  | Nutritional supplements use | 0.772 | -0.017 (-0.614, 0.580) |
|  | No dietary change | 0.845 | 0.008 (-0.750, 0.767) |
| **H-ORAC** | Age | 0.717 | -0.138 (-0.672, 0.397) |
|  | Smoking | 0.860 | 0.000 (-0.560, 0.561) |
|  | Nutritional supplements use | 0.091 | -0.560 (-1.304, 0.184) |
|  | No dietary change | 0.896 | -0.037 (-0.789, 0.715) |
| **L-ORAC** | Age | 0.474 | 0.237 (-0.329, 0.803) |
|  | Smoking | 0.353 | 0.446 (-0.150, 1.041) |
|  | Nutritional supplements use | 0.398 | -0.154 (-0.885, 0.577) |
|  | No dietary change | 0.612 | -0.048 (-0.809, 0.713) |
| **TP** | Age | 0.146 | -0.332 (-0.912, 0.248) |
|  | Smoking | 0.415 | 0.394 (-0.189, 0.977) |
|  | Nutritional supplements use | 0.266 | -0.256 (-1.006, 0.495) |
|  | No dietary change | 0.439 | 0.389 (-0.381, 1.158) |
| **Sperm concentration** | |  |  |
| **FRAP** | Age | 0.427 | 0.173 (-0.343, 0.688) |
|  | Smoking | 0.350 | -0.058 (-0.623, 0.506) |
|  | Nutritional supplements use | 0.728 | 0.236 (-0.419, 0.891) |
|  | No dietary change | 0.690 | 0.299 (-0.450, 1.048) |
| **T-ORAC** | Age | 0.852 | -0.068 (-0.593 0.458) |
|  | Smoking | 0.563 | 0.315 (-0.241, 0.871) |
|  | Nutritional supplements use | 0.106 | -0.329 (-1.075, 0.417) |
|  | No dietary change | 0.720 | 0.029 (-0.702, 0.760) |
| **TRAP** | Age | 0.624 | -0.005 (-0.499, 0.489) |
|  | Smoking | 0.655 | -0.092 (-0.629, 0.445) |
|  | Nutritional supplements use | 0.555 | 0.159 (-0.407, 0.725) |
|  | No dietary change | 0.230 | 0.432 (-0.303, 1.167) |
| **TEAC** | Age | 0.990 | -0.109 (-0.612, 0.395) |
|  | Smoking | 0.832 | -0.018 (-0.557, 0.520) |
|  | Nutritional supplements use | 0.926 | 0.044 (-0.550, 0.638) |
|  | No dietary change | 0.190 | 0.485 (-0.252, 1.221) |
| **H-ORAC** | Age | 0.683 | -0.136 (-0.654 0.382) |
|  | Smoking | 0.970 | 0.126 (-0.423, 0.675) |
|  | Nutritional supplements use | 0.062 | -0.454 (-1.207, 0.298) |
|  | No dietary change | 0.690 | -0.013 (-0.744, 0.718) |
| **L-ORAC** | Age | 0.731 | 0.107 (-0.440, 0.653) |
|  | Smoking | 0.411 | 0.470 (-0.110, 1.050) |
|  | Nutritional supplements use | 0.411 | 0.019 (-0.684, 0.721) |
|  | No dietary change | 0.145 | -0.269 (-1.014, 0.477) |
| **TP** | Age | 0.136 | -0.345 (-0.909, 0.219) |
|  | Smoking | 0.358 | 0.500 (-0.076, 1.075) |
|  | Nutritional supplements use | 0.164 | -0.158 (-0.926, 0.610) |
|  | No dietary change | 0.691 | 0.371 (-0.378, 1.120) |
| **Progressive motility** | |  |  |
| **FRAP** | Age | 0.786 | 0.166 (-0.188, 0.519) |
|  | Smoking | 0.597 | 0.049 (-0.274, 0.371) |
|  | Nutritional supplements use | 0.256 | -0.410 (-0.912, 0.091) |
|  | No dietary change | 0.498 | 0.212 (-0.264, 0.687) |
| **T-ORAC** | Age | 0.485 | 0.333 (-0.019, 0.684) |
|  | Smoking | 0.112 | 0.215 (-0.109, 0.540) |
|  | Nutritional supplements use | 0.971 | -0.144 (-0.526, 0.239) |
|  | No dietary change | 0.188 | 0.349 (-0.103, 0.801) |
| **TRAP** | Age | 0.922 | 0.103 (-0.237, 0.444) |
|  | Smoking | 0.777 | -0.052 (-0.363, 0.259) |
|  | Nutritional supplements use | 0.090 | -0.543 (-1.023, -0.062) |
|  | No dietary change | 0.191 | 0.266 (-0.196, 0.729) |
| **TEAC** | Age | 0.702 | 0.019 (-0.324, 0.362) |
|  | Smoking | 0.798 | -0.063 (-0.373, 0.247) |
|  | Nutritional supplements use | 0.161 | -0.478 (-0.934, -0.022) |
|  | No dietary change | 0.540 | 0.105 (-0.350, 0.560) |
| **H-ORAC** | Age | 0.737 | 0.081 (-0.260, 0.412) |
|  | Smoking | 0.591 | 0.007 (-0.306, 0.319) |
|  | Nutritional supplements use | 0.145 | 0.042 (-0.283, 0.368) |
|  | No dietary change | 0.970 | 0.018 (-0.428, 0.464) |
| **L-ORAC** | Age | 0.824 | 0.201 (-0.148, 0.550) |
|  | Smoking | 0.243 | 0.119 (-0.200, 0.438) |
|  | Nutritional supplements use | 0.982 | -0.177 (-0.549, 0.196) |
|  | No dietary change | 0.471 | 0.187 (-0.260, 0.635) |
| **TP** | Age | 0.200 | 0.399 (0.052, 0.745) |
|  | Smoking | 0.509 | 0.068 (-0.253, 0.389) |
|  | Nutritional supplements use | 0.234 | 0.032 (-0.310, 0.374) |
|  | No dietary change | 0.241 | 0.308 (-0.145, 0.761) |
| **Total motility** | |  |  |
| **FRAP** | Age | 0.587 | 0.284 (-0.068, 0.635) |
|  | Smoking | 0.785 | -0.103 (-0.424, 0.218) |
|  | Nutritional supplements use | 0.064 | -0.474 (-0.992, 0.044) |
|  | No dietary change | 0.375 | 0.238 (-0.233, 0.710) |
| **T-ORAC** | Age | 0.889 | 0.252 (-0.099, 0.602) |
|  | Smoking | 0.298 | 0.103 (-0.220, 0.426) |
|  | Nutritional supplements use | 0.704 | -0.114 (-0.498, 0.269) |
|  | No dietary change | 0.087 | 0.412 (-0.037, 0.862) |
| **TRAP** | Age | 0.790 | 0.143 (-0.197, 0.483) |
|  | Smoking | 0.694 | -0.056 (-0.364, 0.253) |
|  | Nutritional supplements use | 0.082 | -0.455 (-0.915, 0.004) |
|  | No dietary change | 0.308 | 0.198 (-0.257, 0.652) |
| **TEAC** | Age | 0.842 | 0.102 (-0.238, 0.442) |
|  | Smoking | 0.719 | -0.178 (-0.486, 0.129) |
|  | Nutritional supplements use | 0.078 | -0.465 (-0.914, -0.016) |
|  | No dietary change | 0.558 | 0.084 (-0.364, 0.532) |
| **H-ORAC** | Age | 0.618 | 0.062 (-0.277, 0.402) |
|  | Smoking | 0.735 | -0.048 (-0.358, 0.263) |
|  | Nutritional supplements use | 0.238 | 0.079 (-0.247, 0.405) |
|  | No dietary change | 0.808 | 0.051 (-0.388, 0.490) |
| **L-ORAC** | Age | 0.798 | 0.146 (-0.203, 0.495) |
|  | Smoking | 0.357 | 0.062 (-0.256, 0.380) |
|  | Nutritional supplements use | 0.702 | 0.139 (-0.513, 0.236) |
|  | No dietary change | 0.292 | 0.250 (-0.193, 0.693) |
| **TP** | Age | 0.759 | 0.248 (-0.097, 0.593) |
|  | Smoking | 0.754 | -0.017 (-0.335, 0.302) |
|  | Nutritional supplements use | 0.437 | 0.054 (-0.289, 0.397) |
|  | No dietary change | 0.171 | 0.327 (-0.120, 0.774) |
| **Normal sperm morphology** | |  |  |
| **FRAP** | Age | 0.682 | 0.241 (-0.121, 0.603) |
|  | Smoking | 0.323 | 0.133 (-0.213, 0.480) |
|  | Nutritional supplements use | 0.392 | 0.125 (-0.267, 0.517) |
|  | No dietary change | 0.031 | 0.616 (0.092, 1.140) |
| **T-ORAC** | Age | 0.538 | 0.253 (-0.105, 0.611) |
|  | Smoking | 0.649 | 0.038 (-0.305, 0.380) |
|  | Nutritional supplements use | 0.499 | -0.155 (-0.541, 0.231) |
|  | No dietary change | 0.734 | 0.004 (-0.482, 0.490) |
| **TRAP** | Age | 0.876 | 0.147 (-0.210, 0.505) |
|  | Smoking | 0.560 | 0.038 (-0.304, 0.379) |
|  | Nutritional supplements use | 0.324 | 0.114 (-0.258, 0.486) |
|  | No dietary change | 0.064 | 0.494 (-0.013, 1.000) |
| **TEAC** | Age | 0.406 | -0.078 (-0.436, 0.280) |
|  | Smoking | 0.877 | -0.072 (-0.408, 0.265) |
|  | Nutritional supplements use | 0.182 | 0.124 (-0.224, 0.472) |
|  | No dietary change | 0.145 | 0.341 (-0.155, 0.838) |
| **H-ORAC** | Age | 0.823 | 0.063 (-0.288, 0.415) |
|  | Smoking | 0.272 | 0.101 (-0.235, 0.437) |
|  | Nutritional supplements use | 0.756 | -0.130 (-0.502, 0.243) |
|  | No dietary change | 0.474 | 0.202 (-0.281, 0.685) |
| **L-ORAC** | Age | 0.080 | 0.433 (0.071, 0.795) |
|  | Smoking | 0.648 | 0.041 (-0.304, 0.380) |
|  | Nutritional supplements use | 0.742 | -0.094 (-0.473, 0.285) |
|  | No dietary change | 0.402 | -0.108 (-0.596, 0.380) |
| **TP** | Age | 0.926 | 0.134 (-0.221, 0.490) |
|  | Smoking | 0.933 | -0.052 (-0.394, 0.291) |
|  | Nutritional supplements use | 0.189 | -0.274 (-0.680, 0.132) |
|  | No dietary change | 0.571 | 0.208 (-0.275, 0.690) |

FRAP: Ferric-reducing ability of plasma; H-ORAC: Hydrophilic oxygen radical absorbance capacity; L-ORAC: Lipophilic oxygen radical absorbance capacity; RERI: Relative excess risk due to interaction; T-ORAC: Total oxygen radical absorbance capacity; TEAC: Trolox equivalent antioxidant capacity; TP: Total phenolics; TRAP: Total radical-trapping antioxidant parameter.

The model was adjusted for total energy intake (kcal/day), region (Liaoning/other provinces), age (years), BMI (kg/m^2^), fiber intake (g/day), annual family income (RMB; thousand yuan), physical activity (MET/hours/week), abstinence time (days), smoking (yes/no), drinking (yes/no), education (junior secondary or below, senior high school/technical secondary school, and junior college/university or above), occupation (employed/unemployed), nutritional supplements use (yes/no), dietary change (yes/no), and cooking methods (times/week). In additive interaction analyses, when a variable was the analyzed interaction factor, it is removed from the adjustment covariables.

**Supplementary Table S5** Multivariate-adjusted quantile regression coefficients with 95% confidence intervals for semen parameters in relation to the DTAC indices: sensitivity analysis excluding normozoospermic men (n=1132).

| **Variables** | **Ejaculate volume**  **(**mL**)** | **Total sperm count**  **(**10^6^/mL**)** | **Sperm concentration**  **(**10^6^/mL**)** | **Progressive motility**  **(**%**)** | **Total motility**  **(**%**)** | **Normal sperm morphology (**%**)** |
| --- | --- | --- | --- | --- | --- | --- |
| **FRAP (**mmol/d**)** | |  |  |  |  |  |
| T1 | 0 (Ref) | 0 (Ref) | 0 (Ref) | 0 (Ref) | 0 (Ref) | 0 (Ref) |
| T2 | -0.159 (-0.416, 0.098) | 9.782 (-13.172, 32.735) | 2.142 (-3.752, 8.035) | 0.739 (-1.663, 3.142) | 0.889 (-2.087, 3.865) | 0.090 (-0.408, 0.589) |
| T3 | -0.306 (-0.546, -0.066) | 7.606 (-13.564, 28.776) | 3.956 (-1.141, 9.052) | 2.011 (-0.372, 4.394) | 2.181 (-0.803, 5.165) | 0.034 (-0.410, 0.479) |
| *P*-trend | 0.007 | 0.796 | 0.776 | 0.945 | 0.998 | 0.874 |
| Continuous | -0.011 (-0.022, -0.000) | -0.055 (-1.067, 0.957) | 0.034 (-0.239, 0.306) | -0.043 (-0.161, 0.075) | -0.054 (-0.205, 0.098) | -0.006 (-0.026, 0.013) |
| **T-ORAC** **(**μmol TE/d**)** | |  |  |  |  |  |
| T1 | 0 (Ref) | 0 (Ref) | 0 (Ref) | 0 (Ref) | 0 (Ref) | 0 (Ref) |
| T2 | -0.128 (-0.361, 0.105) | 3.100 (-16.693, 22.893) | -0.298 (-5.680, 5.083) | -0.885 (-3.021, 1.250) | -1.575 (-4.316, 1.166) | -0.058 (-0.464, 0.348) |
| T3 | -0.355 (-0.584, -0.126) | 6.908 (-13.003, 26.819) | 2.278 (-2.854, 7.409) | -0.748 (-2.861, 1.366) | -2.259 (-4.824, 0.306) | -0.023 (-0.483, 0.437) |
| *P*-trend | 0.172 | 0.862 | 0.698 | 0.941 | 0.683 | 0.572 |
| Continuous | -0.000 (-0.000, -0.000) | 0.000 (-0.000, 0.001) | 0.000 (-0.000, 0.000) | -0.000 (-0.000, 0.000) | -0.000 (-0.000, -0.000) | 0.000 (-0.000, 0.000) |
| **TRAP (**mmol TE/d**)** | |  |  |  |  |  |
| T1 | 0 (Ref) | 0 (Ref) | 0 (Ref) | 0 (Ref) | 0 (Ref) | 0 (Ref) |
| T2 | 0.013 (-0.207, 0.232) | 9.587 (-9.582, 28.755) | 4.198 (-0.775, 9.170) | 1.248 (-0.913, 3.409) | 1.699 (-0.929, 4.327) | 0.008 (-0.386, 0.401) |
| T3 | -0.256 (-0.478, -0.035) | -4.891 (-25.081, 15.299) | 3.215 (-2.075, 8.504) | -0.697 (-2.859, 1.464) | -1.202 (-3.835, 1.431) | 0.104 (-0.351, 0.560) |
| *P*-trend | 0.002 | 0.234 | 0.697 | 0.100 | 0.055 | 0.832 |
| Continuous | -0.033 (-0.067, 0.000) | 0.352 (-2.514, 3.218) | 0.102 (-0.627, 0.832) | -0.136 (-0.436, 0.164) | -0.242 (-0.606, 0.123) | 0.011 (-0.056, 0.077) |
| **TEAC (**mmol TE/d**)** | |  |  |  |  |  |
| T1 | 0 (Ref) | 0 (Ref) | 0 (Ref) | 0 (Ref) | 0 (Ref) | 0 (Ref) |
| T2 | 0.014 (-0.232, 0.260) | 11.736 (-8.109, 31.581) | 4.225 (-1.221, 9.670) | 0.610 (-1.785, 3.004) | 0.377 (-2.477, 3.232) | -0.049 (-0.499, 0.401) |
| T3 | -0.172 (-0.424, 0.081) | -6.017 (-26.307, 14.274) | 1.593 (-3.806, 6.992) | -0.073 (-2.483, 2.336) | -0.619 (-3.572, 2.335) | 0.066 (-0.422, 0.555) |
| *P*-trend | 0.094 | 0.391 | 0.912 | 0.101 | 0.077 | 0.816 |
| Continuous | -0.054 (-0.103, -0.004) | -0.848 (-5.248, 3.551) | 0.177 (-0.919, 1.273) | -0.369 (-0.840, 0.103) | -0.427 (-1.007, 0.153) | 0.016 (-0.082, 0.114) |
| **H-ORAC** (μmol TE/d) | |  |  |  |  |  |
| T1 | 0 (Ref) | 0 (Ref) | 0 (Ref) | 0 (Ref) | 0 (Ref) | 0 (Ref) |
| T2 | 0.127 (-0.129, 0.382) | 5.968 (-13.516, 25.451) | -2.056 (-7.064, 2.952) | -2.208 (-4.323, -0.092) | -2.871 (-5.516, -0.226) | -0.392 (-0.776, -0.007) |
| T3 | -0.168 (-0.373, 0.037) | 9.582 (-10.285, 29.449) | 1.782 (-3.630, 7.193) | -2.035 (-4.118, 0.048) | -2.424 (-4.914, 0.066) | -0.140 (-0.595, 0.315) |
| *P*-trend | 0.526 | 0.669 | 0.735 | 0.411 | 0.317 | 0.570 |
| Continuous | -0.000 (-0.000, -0.000) | 0.001 (-0.001, 0.001) | 0.000 (-0.000, 0.000) | -0.000 (-0.000, 0.000) | -0.000 (-0.000, -0.000) | 0.000 (-0.000, 0.000) |
| **L-ORAC** (μmol TE/d) | |  |  |  |  |  |
| T1 | 0 (Ref) | 0 (Ref) | 0 (Ref) | 0 (Ref) | 0 (Ref) | 0 (Ref) |
| T2 | -0.178 (-0.422, 0.067) | 2.568 (-18.277, 23.414) | 2.592 (-3.084, 8.268) | 1.482 (-0.700, 3.664) | 1.077 (-1.698, 3.852) | 0.233 (-0.152, 0.618) |
| T3 | -0.254 (-0.498, -0.010) | 2.262 (-17.698, 22.222) | 2.414 (-2.820, 7.648) | -2.188 (-4.353, -0.022) | -3.068 (-5.631, -0.506) | -0.227 (-0.691, 0.236) |
| *P*-trend | 0.207 | 0.657 | 0.612 | 0.320 | 0.420 | 0.738 |
| Continuous | -0.000 (-0.000, 0.000) | 0.000 (-0.000, 0.001) | 0.000 (-0.000, 0.000) | -0.000 (-0.000, 0.000) | -0.000 (-0.000, 0.000) | -0.000 (-0.000, 0.000) |
| **TP** (mg GAE/day) | |  |  |  |  |  |
| T1 | 0 (Ref) | 0 (Ref) | 0 (Ref) | 0 (Ref) | 0 (Ref) | 0 (Ref) |
| T2 | -0.050 (-0.277, 0.177) | -3.539 (-21.495, 14.416) | -2.177 (-6.992, 2.638) | -1.500 (-3.754, 0.754) | -2.628 (-5.440, 0.185) | -0.236 (-0.641, 0.169) |
| T3 | -0.305 (-0.549, -0.060) | 4.089 (-18.147, 26.325) | 0.649 (-4.746, 6.043) | -0.307 (-2.450, 1.836) | -1.533 (-4.262, 1.197) | -0.020 (-0.441, 0.401) |
| *P*-trend | 0.082 | 0.983 | 0.289 | 0.675 | 0.668 | 0.536 |
| Continuous | -0.000 (-0.000, -0.000) | 0.002 (-0.008, 0.013) | 0.000 (-0.002, 0.003) | -0.000 (-0.001, 0.001) | -0.001 (-0.002, 0.000) | 0.000 (-0.000, 0.000) |

DTAC: Dietary total antioxidant capacity; FRAP: Ferric-reducing ability of plasma; GAE: Gallic acid equivalents; H-ORAC: Hydrophilic oxygen radical absorbance capacity; L-ORAC: Lipophilic oxygen radical absorbance capacity; Ref: Reference; T: Tertile; T-ORAC: Total oxygen radical absorbance capacity; TE: Trolox equivalents; TEAC: Trolox equivalent antioxidant capacity; TP: Total phenolics; TRAP: Total radical-trapping antioxidant parameter.

The model was adjusted for total energy intake (kcal/day), region (Liaoning/other provinces), age (years), BMI (kg/m^2^), fiber intake (g/day), annual family income (RMB; thousand yuan), physical activity (MET/hours/week), abstinence time (days), smoking (yes/no), drinking (yes/no), education (junior secondary or below, senior high school/technical secondary school, and junior college/university or above), occupation (employed/unemployed), nutritional supplements use (yes/no), dietary change (yes/no), and cooking methods (times/week).

**Supplementary Table S6** Multivariable-adjusted odds ratios with 95% confidence intervals for tertiles of semen parameters in relation to the DTAC indices.

|  | **Ejaculate volume** | | **Total sperm count** | | **Ejaculate volume** | |
| --- | --- | --- | --- | --- | --- | --- |
|  | **T2 vs. T1** | **T3 vs. T1** | **T2 vs. T1** | **T3 vs. T1** | **T2 vs. T1** | **T3 vs. T1** |
| **FRAP** (mmol/d) |  |  |  |  |  |  |
| T1 | Ref | Ref | Ref | Ref | Ref | Ref |
| T2 | 0.860 (0.623, 1.187) | 0.918 (0.667, 1.264) | 0.986 (0.720, 1.350) | 1.029 (0.752, 1.407) | 0.902 (0.661, 1.230) | 0.928 (0.678, 1.272) |
| T3 | 0.870 (0.637, 1.188) | 0.777 (0.566, 1.066) | 1.183 (0.858, 1.632) | 1.047 (0.757, 1.448) | 0.989 (0.721, 1.356) | 1.093 (0.795, 1.503) |
| *P*-trend | 0.815 | 0.144 | 0.804 | 0.940 | 0.773 | 0.510 |
| Continuous | 0.993 (0.978, 1.008) | 0.984 (0.969, 1.000) | 1.007 (0.992, 1.022) | 0.993 (0.977, 1.010) | 1.007 (0.992, 1.023) | 1.005 (0.989, 1.021) |
| **T-ORAC** (μmol TE/d) | |  |  |  |  |  |
| T1 | Ref | Ref | Ref | Ref | Ref | Ref |
| T2 | 0.887 (0.644, 1.221) | 0.894 (0.659, 1.214) | 0.954 (0.699, 1.303) | 1.097 (0.812, 1.483) | 1.010 (0.741, 1.378) | 1.077 (0.797, 1.454) |
| T3 | 0.913 (0.670, 1.242) | 0.729 (0.539, 0.985) | 0.900 (0.656, 1.233) | 1.010 (0.740, 1.377) | 0.987 (0.724, 1.347) | 1.005 (0.743, 1.360) |
| *P*-trend | 0.425 | 0.068 | 0.690 | 0.872 | 0.681 | 0.997 |
| Continuous | 1.000 (1.000, 1.000) | 1.000 (1.000, 1.000) | 1.000 (1.000, 1.000) | 1.000 (1.000, 1.000) | 1.000 (1.000, 1.000) | 1.000 (1.000, 1.000) |
| **TRAP** (mmol TE/d) |  |  |  |  |  |  |
| T1 | Ref | Ref | Ref | Ref | Ref | Ref |
| T2 | 0.996 (0.729, 1.360) | 0.802 (0.589, 1.091) | 0.902 (0.673, 1.234) | 0.764 (0.566, 1.031) | 1.163 (0.861, 1.571) | 1.011 (0.748, 1.365) |
| T3 | 0.873 (0.644, 1.181) | 0.637 (0.470, 0.861) | 1.175 (0.861, 1.606) | 0.865 (0.633, 1.181) | 1.130 (0.834, 1.530) | 0.994 (0.734, 1.346) |
| *P*-trend | 0.123 | 0.003 | 0.072 | 0.226 | 0.910 | 0.849 |
| Continuous | 0.973 (0.936, 1.010) | 0.967 (0.930, 1.004) | 0.991 (0.953, 1.030) | 1.004 (0.967, 1.043) | 1.010 (0.971, 1.050) | 1.022 (0.984, 1.063) |
| **TEAC** (mmol TE/d) |  |  |  |  |  |  |
| T1 | Ref | Ref | Ref | Ref | Ref | Ref |
| T2 | 1.023 (0.748, 1.400) | 0.975 (0.712, 1.335) | 1.020 (0.752, 1.382) | 0.758 (0.554, 1.034) | 0.980 (0.724, 1.327) | 0.995 (0.729, 1.357) |
| T3 | 1.101 (0.813, 1.492) | 0.829 (0.606, 1.133) | 1.110 (0.810, 1.523) | 0.918 (0.667, 1.262) | 0.975 (0.719, 1.324) | 0.991 (0.725, 1.357) |
| *P*-trend | 0.844 | 0.173 | 0.058 | 0.494 | 0.986 | 0.974 |
| Continuous | 0.958 (0.904, 1.014) | 0.947 (0.893, 1.002) | 0.991 (0.936, 1.050) | 0.998 (0.941, 1.058) | 1.019 (0.961, 1.081) | 1.035 (0.977, 1.097) |
| **H-ORAC** (μmol TE/d) | |  |  |  |  |  |
| T1 | Ref | Ref | Ref | Ref | Ref | Ref |
| T2 | 0.817 (0.603, 1.106) | 1.062 (0.789, 1.430) | 1.052 (0.784, 1.411) | 1.169 (0.871, 1.571) | 1.152 (0.859, 1.547) | 1.102 (0.821, 1.481) |
| T3 | 1.047 (0.783, 1.399) | 0.872 (0.646, 1.176) | 1.095 (0.811, 1.479) | 1.107 (0.816, 1.503) | 1.032 (0.768, 1.386) | 0.954 (0.709, 1.283) |
| *P*-trend | 0.787 | 0.423 | 0.309 | 0.495 | 0.482 | 0.785 |
| Continuous | 1.000 (1.000, 1.000) | 1.000 (1.000, 1.000) | 1.000 (1.000, 1.000) | 1.000 (1.000, 1.000) | 1.000 (1.000, 1.000) | 1.000 (1.000, 1.000) |
| **L-ORAC** (μmol TE/d) | |  |  |  |  |  |
| T1 | Ref | Ref | Ref | Ref | Ref | Ref |
| T2 | 0.962 (0.696, 1.329) | 0.872 (0.632, 1.203) | 1.022 (0.743, 1.405) | 1.120 (0.819, 1.533) | 1.197 (0.872, 1.644) | 1.162 (0.849, 1.591) |
| T3 | 0.902 (0.658, 1.236) | 0.805 (0.587, 1.103) | 1.121 (0.813, 1.547) | 0.994 (0.717, 1.378) | 1.233 (0.898, 1.695) | 1.107 (0.806, 1.521) |
| *P*-trend | 0.496 | 0.219 | 0.570 | 0.747 | 0.236 | 0.287 |
| Continuous | 1.000 (1.000, 1.000) | 1.000 (1.000, 1.000) | 1.000 (1.000, 1.000) | 1.000 (1.000, 1.000) | 1.000 (1.000, 1.000) | 1.000 (1.000, 1.000) |
| **TP** (mg GAE/d) |  |  |  |  |  |  |
| T1 | Ref | Ref | Ref | Ref | Ref | Ref |
| T2 | 0.837 (0.617, 1.135) | 1.009 (0.744, 1.368) | 0.973 (0.723, 1.309) | 1.030 (0.763, 1.392) | 1.012 (0.753, 1.360) | 0.927 (0.687, 1.251) |
| T3 | 0.885 (0.660, 1.186) | 0.778 (0.575, 1.052) | 0.855 (0.631, 1.158) | 0.885 (0.650, 1.204) | 0.927 (0.688, 1.250) | 0.903 (0.669, 1.219) |
| *P*-trend | 0.977 | 0.108 | 0.866 | 0.400 | 0.644 | 0.493 |
| Continuous | 1.000 (1.000, 1.000) | 1.000 (1.000, 1.000) | 1.000 (1.000, 1.000) | 1.000 (1.000, 1.000) | 1.000 (1.000, 1.000) | 1.000 (1.000, 1.000) |

DTAC: Dietary total antioxidant capacity; FRAP: Ferric-reducing ability of plasma; GAE: Gallic acid equivalents; H-ORAC: Hydrophilic oxygen radical absorbance capacity; L-ORAC: Lipophilic oxygen radical absorbance capacity; Ref: Reference; T: Tertile; T-ORAC: Total oxygen radical absorbance capacity; TE: Trolox equivalents; TEAC: Trolox equivalent antioxidant capacity; TP: Total phenolics; TRAP: Total radical-trapping antioxidant parameter.

The model was adjusted for total energy intake (kcal/day), region (Liaoning/other provinces), age (years), BMI (kg/m^2^), fiber intake (g/day), annual family income (RMB; thousand yuan), physical activity (MET/hours/week), abstinence time (days), smoking (yes/no), drinking (yes/no), education (junior secondary or below, senior high school/technical secondary school, and junior college/university or above), occupation (employed/unemployed), nutritional supplements use (yes/no), dietary change (yes/no), and cooking methods (times/week).

**Supplementary Table S6 (*Continued*)** Multivariable-adjusted odds ratios with 95% confidence intervals for tertiles of semen parameters in relation to the DTAC indices.

|  | **Progressive motility** | | **Total motility** | | **Normal sperm morphology** | |
| --- | --- | --- | --- | --- | --- | --- |
|  | **T2 vs. T1** | **T3 vs. T1** | **T2 vs. T1** | **T3 vs. T1** | **T2 vs. T1** | **T3 vs. T1** |
| **FRAP** (mmol/d) |  |  |  |  |  |  |
| T1 | Ref | Ref | Ref | Ref | Ref | Ref |
| T2 | 1.105 (0.807, 1.512) | 1.307 (0.957, 1.787) | 1.056 (0.771, 1.445) | 1.264 (0.926, 1.728) | 1.115 (0.814, 1.526) | 0.981 (0.717, 1.343) |
| T3 | 1.117 (0.817, 1.528) | 1.097 (0.798, 1.511) | 1.036 (0.759, 1.415) | 0.965 (0.702, 1.326) | 1.045 (0.764, 1.431) | 0.916 (0.667, 1.257) |
| *P*-trend | 0.090 | 0.693 | 0.116 | 0.736 | 0.680 | 0.459 |
| Continuous | 0.998 (0.983, 1.012) | 0.998 (0.983, 1.013) | 1.003 (0.988, 1.017) | 0.994 (0.979, 1.010) | 1.001 (0.986, 1.016) | 1.000 (0.985, 1.016) |
| **T-ORAC** (μmol TE/d) | |  |  |  |  |  |
| T1 | Ref | Ref | Ref | Ref | Ref | Ref |
| T2 | 0.826 (0.608, 1.123) | 0.806 (0.597, 1.086) | 0.765 (0.562, 1.040) | 0.837 (0.621, 1.127) | 1.102 (0.808, 1.501) | 1.098 (0.813, 1.483) |
| T3 | 0.923 (0.675, 1.263) | 0.849 (0.626, 1.151) | 0.921 (0.674, 1.257) | 0.843 (0.622, 1.143) | 0.913 (0.670, 1.246) | 0.921 (0.682, 1.244) |
| *P*-trend | 0.129 | 0.314 | 0.141 | 0.294 | 0.498 | 0.541 |
| Continuous | 1.000 (1.000, 1.000) | 1.000 (1.000, 1.000) | 1.000 (1.000, 1.000) | 1.000 (1.000, 1.000) | 1.000 (1.000, 1.000) | 1.000 (1.000, 1.000) |
| **TRAP** (mmol TE/d) |  |  |  |  |  |  |
| T1 | Ref | Ref | Ref | Ref | Ref | Ref |
| T2 | 1.065 (0.786, 1.444) | 0.860 (0.638, 1.160) | 1.109 (0.817, 1.507) | 1.023 (0.760, 1.378) | 0.921 (0.682, 1.244) | 0.897 (0.663, 1.212) |
| T3 | 1.073 (0.791, 1.455) | 0.840 (0.620, 1.138) | 1.091 (0.808, 1.474) | 0.740 (0.546, 1.003) | 1.020 (0.753, 1.381) | 1.055 (0.779, 1.431) |
| *P*-trend | 0.236 | 0.184 | 0.997 | 0.028 | 0.505 | 0.723 |
| Continuous | 0.995 (0.959, 1.031) | 0.994 (0.957, 1.033) | 0.995 (0.960, 1.032) | 0.986 (0.949, 1.024) | 1.009 (0.971, 1.048) | 1.014 (0.976, 1.054) |
| **TEAC** (mmol TE/d) |  |  |  |  |  |  |
| T1 | Ref | Ref | Ref | Ref | Ref | Ref |
| T2 | 1.080 (0.797, 1.465) | 0.890 (0.653, 1.213) | 1.103 (0.812, 1.498) | 0.996 (0.731, 1.357) | 0.837 (0.618, 1.134) | 0.793 (0.579, 1.083) |
| T3 | 0.976 (0.718, 1.327) | 0.890 (0.651, 1.215) | 0.900 (0.664, 1.220) | 0.763 (0.558, 1.041) | 0.826 (0.608, 1.121) | 0.909 (0.665, 1.243) |
| *P*-trend | 0.376 | 0.433 | 0.878 | 0.081 | 0.171 | 0.667 |
| Continuous | 0.980 (0.927, 1.037) | 0.990 (0.935, 1.049) | 0.985 (0.931, 1.040) | 0.981 (0.926, 1.039) | 1.006 (0.950, 1.066) | 1.024 (0.966, 1.085) |
| **H-ORAC** (μmol TE/d) | |  |  |  |  |  |
| T1 | Ref | Ref | Ref | Ref | Ref | Ref |
| T2 | 0.765 (0.570, 1.026) | 0.794 (0.593, 1.063) | 0.784 (0.585, 1.050) | 0.800 (0.598, 1.071) | 1.132 (0.845, 1.517) | 0.985 (0.732, 1.326) |
| T3 | 1.075 (0.799, 1.446) | 0.909 (0.672, 1.227) | 1.016 (0.756, 1.365) | 0.920 (0.682, 1.240) | 0.889 (0.661, 1.194) | 0.978 (0.728, 1.312) |
| *P*-trend | 0.096 | 0.589 | 0.111 | 0.614 | 0.993 | 0.814 |
| Continuous | 1.000 (1.000, 1.000) | 1.000 (1.000, 1.000) | 1.000 (1.000, 1.000) | 1.000 (1.000, 1.000) | 1.000 (1.000, 1.000) | 1.000 (1.000, 1.000) |
| **L-ORAC** (μmol TE/d) | |  |  |  |  |  |
| T1 | Ref | Ref | Ref | Ref | Ref | Ref |
| T2 | 1.209 (0.882, 1.660) | 0.703 (0.514, 0.960) | 1.085 (0.791, 1.488) | 0.698 (0.511, 0.952) | 1.172 (0.853, 1.611) | 1.162 (0.850, 1.589) |
| T3 | 1.474 (1.067, 2.041) | 0.793 (0.576, 1.089) | 1.362 (0.988, 1.880) | 0.742 (0.540, 1.018) | 1.258 (0.917, 1.727) | 0.851 (0.620, 1.168) |
| *P*-trend | 0.342 | 0.889 | 0.187 | 0.717 | 0.265 | 0.982 |
| Continuous | 1.000 (1.000, 1.000) | 1.000 (1.000, 1.000) | 1.000 (1.000, 1.000) | 1.000 (1.000, 1.000) | 1.000 (1.000, 1.000) | 1.000 (1.000, 1.000) |
| **TP** (mg GAE/d) |  |  |  |  |  |  |
| T1 | Ref | Ref | Ref | Ref | Ref | Ref |
| T2 | 0.788 (0.586, 1.058) | 0.903 (0.672, 1.214) | 0.781 (0.581, 1.049) | 0.862 (0.642, 1.158) | 1.084 (0.808, 1.456) | 0.994 (0.736, 1.342) |
| T3 | 1.083 (0.805, 1.458) | 0.937 (0.690, 1.272) | 1.051 (0.782, 1.141) | 0.873 (0.644, 1.183) | 0.907 (0.674, 1.221) | 0.963 (0.714, 1.299) |
| *P*-trend | 0.428 | 0.727 | 0.275 | 0.428 | 0.993 | 0.764 |
| Continuous | 1.000 (1.000, 1.000) | 1.000 (1.000, 1.000) | 1.000 (1.000, 1.000) | 1.000 (1.000, 1.000) | 1.000 (1.000, 1.000) | 1.000 (1.000, 1.000) |

DTAC: Dietary total antioxidant capacity; FRAP: Ferric-reducing ability of plasma; GAE: Gallic acid equivalents; H-ORAC: Hydrophilic oxygen radical absorbance capacity; L-ORAC: Lipophilic oxygen radical absorbance capacity; Ref: Reference; T: Tertile; T-ORAC: Total oxygen radical absorbance capacity; TE: Trolox equivalents; TEAC: Trolox equivalent antioxidant capacity; TP: Total phenolics; TRAP: Total radical-trapping antioxidant parameter.

The model was adjusted for total energy intake (kcal/day), region (Liaoning/other provinces), age (years), BMI (kg/m^2^), fiber intake (g/day), annual family income (RMB; thousand yuan), physical activity (MET/hours/week), abstinence time (days), smoking (yes/no), drinking (yes/no), education (junior secondary or below, senior high school/technical secondary school, and junior college/university or above), occupation (employed/unemployed), nutritional supplements use (yes/no), dietary change (yes/no), and cooking methods (times/week).

**Supplementary Table S7** Multivariate-adjusted linear regression coefficients with 95% CIs for semen parameters in relation to DTAC indices after multiple imputation of missing data.

| **Variables** | **Ejaculate volume (**mL**)** | **Total sperm count (**10^6^/mL**)** | **Sperm concentration (**10^6^/mL**)** | **Progressive motility (**%**)** | **Progressive motility (**%**)** | **Normal sperm morphology (**%**)** |
| --- | --- | --- | --- | --- | --- | --- |
| **FRAP** (mmol/d) | |  |  |  |  |  |
| T1 | Ref | Ref | Ref | Ref | Ref | Ref |
| T2 | -0.153 (-0.323, 0.018) | 1.740 (-14.274, 17.754) | 0.452 (-4.349, 5.253) | 1.199 (-0.620, 3.018) | 0.961 (-1.247, 3.169) | 0.292 (-0.085, 0.668) |
| T3 | -0.193 (-0.366, -0.021) | -6.256 (-22.516, 10.005) | -0.800 (-5.665, 4.068) | 0.355 (-1.485, 2.196) | 0.348 (-1.886, 2.583) | 0.213 (-0.172, 0.599) |
| *P*-trend | 0.049 | 0.281 | 0..567 | 0.872 | 0.934 | 0.422 |
| Continuous | -0.009 (-0.018, -0.001) | -0.434 (-1.226, 0.358) | -0.051 (-0.289, 0.187) | -0.024 (-0.115, 0.067) | -0.019 (-0.129, 0.091) | 0.007 (-0.011, 0.026) |
| **T-ORAC** (μmol TE/d) | |  |  |  |  |  |
| T1 | Ref | Ref | Ref | Ref | Ref | Ref |
| T2 | 0.049 (-0.121, 0.218) | -3.521 (-19.452, 12.411) | -0.443 (-5.216, 4.330) | -0.913 (-2.723, 0.897) | -0.606 (-2.803, 1.591) | -0.200 (-0.584, 0.184) |
| T3 | -0.152 (-0.318, 0.014) | 0.979 (-14.652, 16.611) | 2.905 (-1.775, 7.585) | -1.268 (-3.034, 0.499) | -1.176 (-3.322, 0.969) | -0.187 (-0.573, 0.199) |
| *P*-trend | 0.157 | 0.859 | 0.431 | 0.113 | 0.239 | 0.219 |
| Continuous | -0.000 (-0.000, 0.000) | 0.000 (-0.000, 0.000) | 0.000 (-0.000, 0.000) | -0.000 (-0.000, 0.000) | -0.000 (-0.000, 0.000) | -0.000 (-0.000, 0.000) |
| **TRAP** (mmol TE/d) | |  |  |  |  |  |
| T1 | Ref | Ref | Ref | Ref | Ref | Ref |
| T2 | -0.191 (-0.356, -0.025) | 7.979 (-7.603, 23.560) | 5.602 (0.932, 10.272) | -0.169 (-1.940, 1.602) | 0.166 (-1.984, 2.315) | 0.155 (-0.219, 0.530) |
| T3 | -0.310 (-0.477, -0.143) | -10.881 (-26.620, 4.859) | 0.448 (-4.271, 5.167) | -1.486 (-3.274, 0.303) | -1.507 (-3.678, 0.664) | 0.199 (-0.173, 0.572) |
| *P*-trend | < 0.001 | 0.079 | 0.727 | 0.079 | 0.123 | 0.338 |
| Continuous | -0.026 (-0.047, -0.006) | 0.025 (-1.912, 1.962) | 0.219 (-0.362, 0.800) | -0.059 (-0.280, 0.163) | -0.002 (-0.270, 0.266) | 0.038 (-0.009, 0.085) |
| **TEAC** (mmol TE/d) | |  |  |  |  |  |
| T1 | Ref | Ref | Ref | Ref | Ref | Ref |
| T2 | -0.108 (-0.274, 0.059) | 1.584 (-14.063, 17.232) | 2.936 (-1.755, 7.626) | -0.098 (-1.877, 1.681) | -0.019 (-2.178, 2.139) | -0.137 (-0.514, 0.241) |
| T3 | -0.225 (-0.397, -0.053) | -11.172 (-27.385, 5.042) | -1.205 (-6.071, 3.660) | -1.004 (-2.850, 0.841) | -1.012 (-3.251, 1.228) | 0.049 (-0.342, 0.440) |
| *P*-trend | 0.008 | 0.111 | 0.409 | 0.234 | 0.311 | 0.616 |
| Continuous | -0.042 (-0.073, -0.011) | -0.276 (-3.213, 2.661) | 0.331 (-0.551, 1.213) | -0.107 (-0.444, 0.230) | -0.015 (-0.423, 0.394) | 0.058 (-0.012, 0.129) |
| **H-ORAC** (μmol TE/d) | |  |  |  |  |  |
| T1 | Ref | Ref | Ref | Ref | Ref | Ref |
| T2 | 0.071 (-0.090, 0.233) | 9.644 (-5.550, 24.838) | 2.493 (-2.057, 7.045) | -0.072 (-1.792, 1.648) | 0.259 (-1.830, 2.348) | -0.091 (-0.455, 0.272) |
| T3 | -0.069 (-0.232, 0.095) | 4.171 (-11.207, 19.550) | 3.544 (-1.062, 8.150) | -1.307 (-3.047, 0.433) | -1.052 (-3.166, 1.061) | -0.079 (-0.454, 0.296) |
| *P*-trend | 0.386 | 0.652 | 0.176 | 0.117 | 0.286 | 0.546 |
| Continuous | -0.000 (-0.000, 0.000) | 0.000 (-0.000, 0.001) | 0.000 (-0.000, 0.000) | -0.000 (-0.000, 0.000) | -0.000 (-0.000, 0.000) | -0.000 (-0.000, 0.000) |
| **L-ORAC** (μmol TE/d) | |  |  |  |  |  |
| T1 | Ref | Ref | Ref | Ref | Ref | Ref |
| T2 | -0.022 (-0.196, 0.152) | 2.472 (-13.859, 18.804) | 1.579 (-3.314, 6.472) | 1.486 (-0.359, 3.332) | 2.193 (-0.047, 4.433) | 0.164 (-0.221, 0.549) |
| T3 | -0.138 (-0.311, 0.034) | -3.291 (-19.541, 12.959) | 1.735 (-3.132, 6.602) | -1.367 (-3.202, 0.468) | -1.502 (-3.729, 0.724) | -0.227 (-0.613, 0.160) |
| *P*-trend | 0.239 | 0.905 | 0.430 | 0.836 | 0.954 | 0.638 |
| Continuous | -0.000 (-0.000, 0.000) | 0.000 (-0.000, 0.000) | 0.000 (-0.000, 0.000) | -0.000 (-0.000, 0.000) | -0.000 (-0.000, 0.000) | -0.000 (-0.000, 0.000) |
| **TP** (mg GAE/d) | |  |  |  |  |  |
| T1 | Ref | Ref | Ref | Ref | Ref | Ref |
| T2 | -0.072 (-0.235, 0.091) | -4.774 (-20.136, 10.587) | 1.294 (-3.305, 5.892) | -0.032 (-1.768, 1.704) | 0.344 (-1.763, 2.452) | -0.195 (-0.560, 0.170) |
| T3 | -0.093 (-0.260, 0.074) | -4.810 (-20.521, 10.902) | 0.765 (-3.942, 5.471) | -0.811 (-2.588, 0.965) | -0.910 (-3.067, 1.246) | -0.141 (-0.536, 0.254) |
| *P*-trend | 0.229 | 0.480 | 0.772 | 0.361 | 0.414 | 0.312 |
| Continuous | -0.000 (-0.000, 0.000) | -0.002 (-0.009, 0.005) | 0.000 (-0.002, 0.002) | -0.000 (-0.001, 0.000) | -0.000 (-0.001, 0.000) | -0.000 (-0.000, 0.000) |

Dietary total antioxidant capacity; FRAP: Ferric-reducing ability of plasma; GAE: Gallic acid equivalents; H-ORAC: Hydrophilic oxygen radical absorbance capacity; L-ORAC: Lipophilic oxygen radical absorbance capacity; Ref: Reference; T: Tertile; T-ORAC: Total oxygen radical absorbance capacity; TE: Trolox equivalents; TEAC: Trolox equivalent antioxidant capacity; TP: Total phenolics; TRAP: Total radical-trapping antioxidant parameter.

The model was adjusted for total energy intake (kcal/day), region (Liaoning/other provinces), age (years), BMI (kg/m^2^), fiber intake (g/day), annual family income (RMB; thousand yuan), physical activity (MET/hours/week), abstinence time (days), smoking (yes/no), drinking (yes/no), education (junior secondary or below, senior high school/technical secondary school, and junior college/university or above), occupation (employed/unemployed), nutritional supplements use (yes/no), dietary change (yes/no), and cooking methods (times/week).
