# Supplementary material for: Association between dietary total antioxidant capacity and semen quality among men attending an infertility clinic: a cross-sectional study
Source: Hum Reprod Open. 2023 Oct 31;2023(4):hoad041. doi: 10.1093/hropen/hoad041 (PMC10639034; doi:10.1093/hropen/hoad041)
Supplement: hoad041_Supplementary_Figures [file hoad041_supplementary_figures.docx]

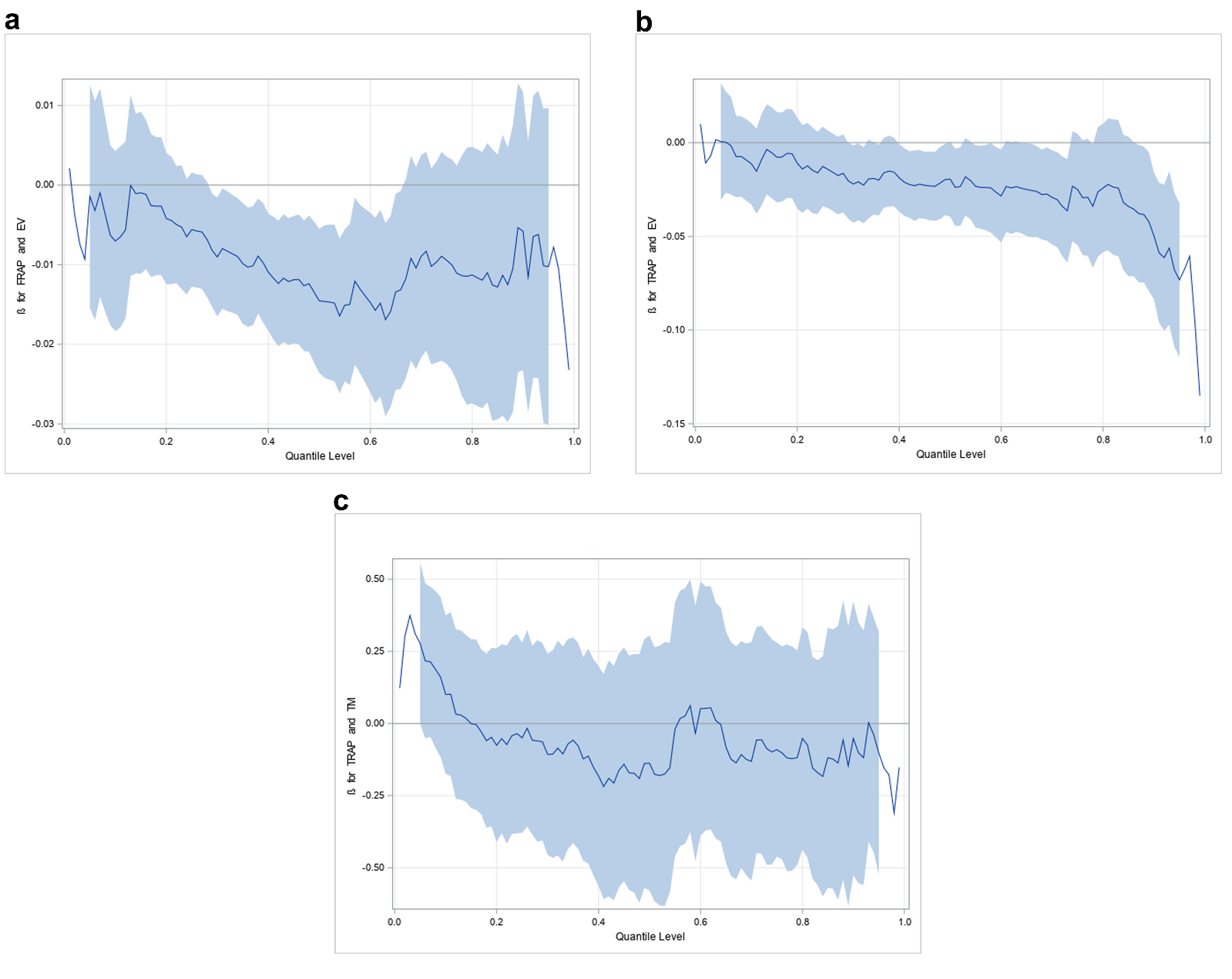


**Supplementary Figure S1** The statistically significant quantile regression results.

The data presented are for (**a**) ferric-reducing ability of plasma and ejaculate volume, (**b**) total radical-trapping antioxidant parameter and ejaculate volume, and (**c**) total radical-trapping antioxidant parameter and total motility. EV: Ejaculate volume; FRAP: Ferric-reducing ability of plasma; TM: Total motility; TRAP: Total radical-trapping antioxidant parameter.


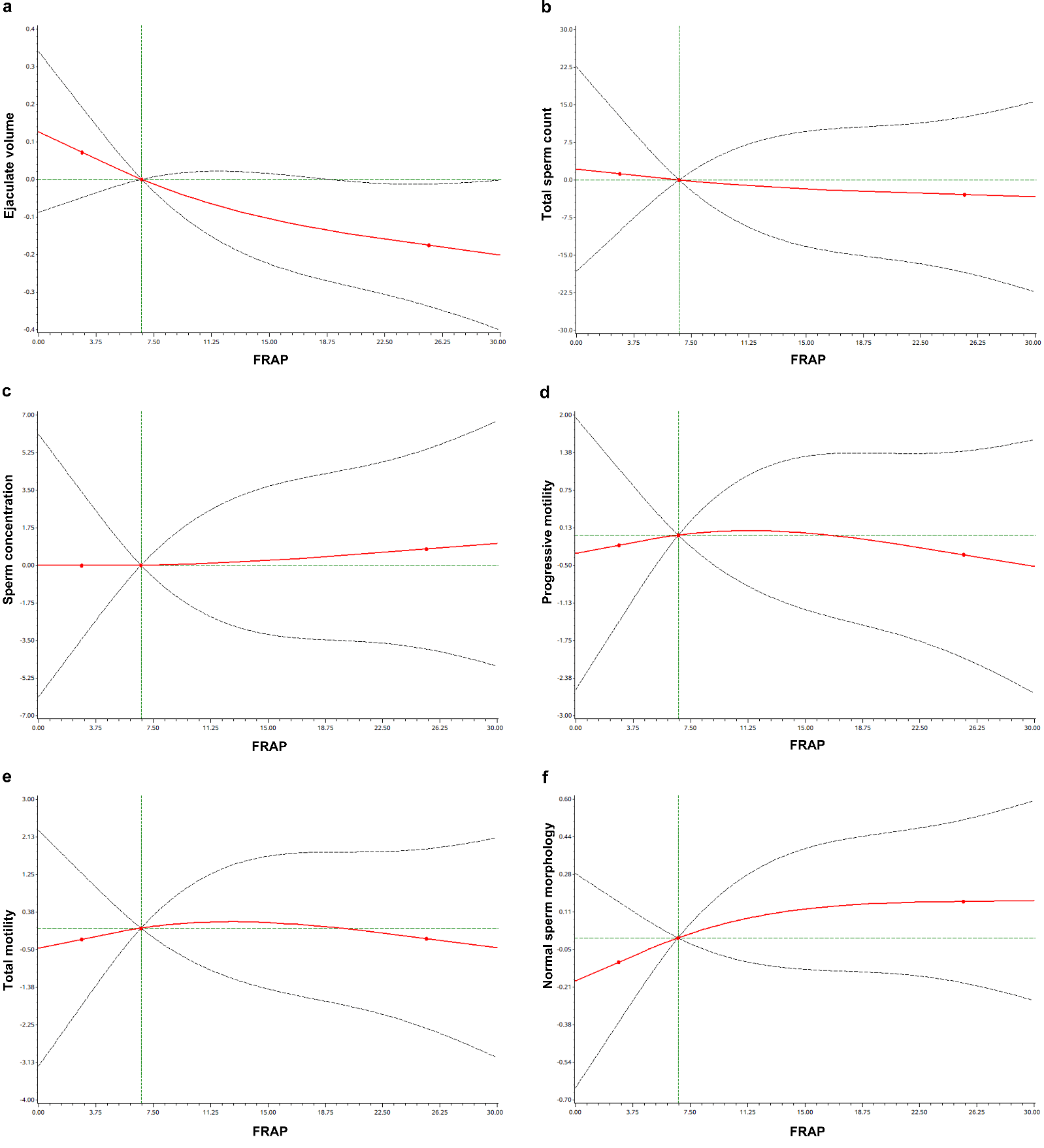


**Supplementary Figure S2** The restricted cubic spline curves for ferric-reducing ability of plasma and semen quality parameters.

The restricted cubic spline curves for ferric-reducing ability of plasma and (**a**) ejaculate volume, (**b**) total sperm count, (**c**) sperm concentration, (**d**) progressive motility, (**e**) total motility, and (**f**) normal sperm morphology. FRAP: Ferric-reducing ability of plasma.


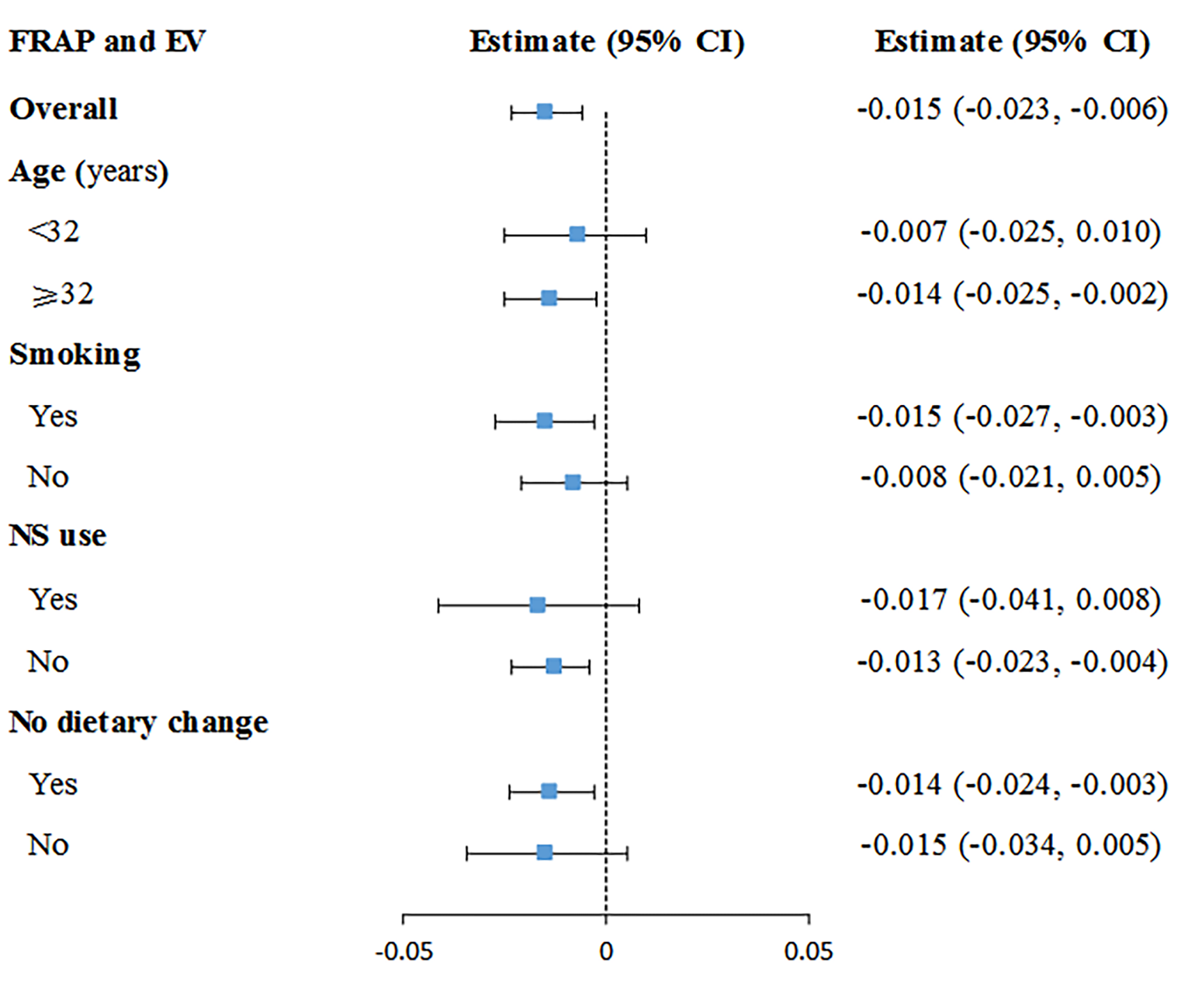


**Supplementary Figure S3** Subgroup analysis results for ferric-reducing ability of plasma and ejaculate volume.

EV: Ejaculate volume; FRAP: Ferric-reducing ability of plasma. NS: Nutritional supplements.
